# Chapter I: Mind and Disposition: Fixed—Wrong

**Published:** 1889

**Authors:** Edmund J. Lee


**Fixed** notions. See Delusions.
**Fond.** See Affectionate.
**Foolish** behavior: acon. all-c. anac. arg-n. *bell.* cann-i. carb-an. carb-v. cic. con. croc. cupr. *hyos.* ign. lach. merc. nx-m. nx-v. op. par. phys. secale. seneg. STRAM. tanac. *verat.*
— — See also Childish.
— — **morning**, on waking: aur.
— — **night**: cic.
— — during **spasms**: secale.
**Forgetful**: *acon.* agar. *agn.* alum. am-c. *anac. arg. arn.* ars. arum-t. aur. bar. bell. bov. brom. bry. calad. calc. calc-p. camph. cann-i. cann-s. canth. caps. carb-an. carb-v. cham. chel. chin. cic. cinnb. cocc. colch. *con.* creos. croc. cupr. cyc. dig. fluor-ac. graph. guai. gymn. ham. hell. hepar. hipp. hydr. hyos. ign. k-ca. lac-can. *lach.* laur. lyc. mag-c. merc. mezer. mosch. naja. nat-c. nat-m. nitr. nit-ac. nx-m. nx-v. olnd. op. petr. phos. ph-ac. plat. puls. ran-b. rheum. rhod. rhus. ruta. sabin. secale. selen. sep. sil. spig. stann. staph. stram. stront. *sulph.* tabac. tarax. thu. verat. verb. viol-od. *znc.* zing.
— See Mind, absence of; Memory, lost; Confused, Mistakes, etc.
— of past **actions**: calc-p. camph. chel. fluor-ac. lach.
— — **business**: chel. selen. tellur.
— — **going** to do, of what he was: bell. calc-p. *chel.* creos. hydr.
— — **dates**, of: acon. fluor-ac.
— — **names**: anac. chin-s. chlor. *guai.* lach. lith. lyc. merc. olnd. rhus. *sulph.*
— — — of his own: k-bro. sulph.
— — — his personal identity is confused: alum. valer.
— — purchases, goes off and leaves them: agn. lac-can.
— — **reading**: lyc.
— — **said**, of what he has: cann-i. mur-ac. nitr. stram.
— — **shaving** or dressing: chel.
— — **streets**, of well-known: *glon.* petr. nx-m.
— — **time** and place: merc.
— — **wind**, watch, to: fluor-ac.
— — **words**: *arn.* bar. benz-ac. *cann-i.* carb-v. ham. hydr. *k-bro. lach.* lyc. nx-v. podo. rhod.
— — — when speaking: *arn.* bar-ac.
— — **write**, of what he is about to: croc.
— in morning: phos.
— on waking: stann.
— — when he is half awake, yet has distinct recollection when half asleep: selen.
**Forgotten** something, feels constantly as if he had: iod.
**Forsaken** feeling: bar. camph. carb-an. hura. lam. mag-m. nat-c. plat. psor. puls. rhus.
— — on waking: lach.
— sensation of isolation: anac. cann-i. coca.
**Frantic**, frenzy. See Rage.
**Fretfulness**, irritability, peevishness: abrot. acet-ac. acon. æsc-h. æth. agar. agn. am-c. *am-m.* anac. ang. ant-c. ant-t. apis. arn. *ars.* asar. aur. bar. bell. benz-ac. berb. bism. borax. bov. *bry. calc.* calc-p. cann-s. canth. caps. carb-an. *carb-v. caus. cham.* chel. chin. cimic. *cina.* cinnb. clem. cocc. colch. coloc. con. coral. *creos.* croc. cupr. cyc. dros. eugen. *evon.* ferr. gamb. gels. *graph. grat.* ham. hell. hydr-ac. *ign.* indg. ipec. k-ca. k-iod. lac-can. lach. lact. *led.* lil-t. lyc. mag-c. mag-m. *mang. marum.* merc. mezer. mur-ac. nat-c. nat-m. nat-s. *nit-ac.* nitr. *nx-v.* olnd. op. par. petr. phel. *phos. ph-ac.* plat. *puls. ran-b.* rheum. *rhus.* sabad. sabin. samb. sars. seneg. *sep. sil.* spig. spong. squil. stann. *staph.* stram. *stront.* sulph. *sul-ac.* thea. tong. ustil. verat. verb. *znc.* zing.
— Compare with Ill-humor.
— **alternating** with cheerfulness: aur.
— — with indifference: sep.
— about trifles: meph. nat-m. nit-ac. squil.
— — himself: bell.
— afternoon: æth.
— evening: mag-c. puls. *znc.*
— morning: am-c. am-m. nat-s. nx-v.
— open air, in: æth. sabin.
— bad effects from: cist.
— consolation, aggr.: hell.
— from music: caus.
— nervous: chin.
— pains, during the: chin. coff. nx-v. phos.
— during hot stage of fever: acon. anac. ars. carb-v. bry. *cham.* lach. phos. ph-ac. plan.
— during chilly stage: ars. bell. borax. caus. mezer. phos. puls.
— after seminal emissions: selen.

— during sweat: mag-c.
— on waking, children: lyc.
— stool, before: borax.
**Frightened** easily: *acon.* alum. ang. ant-c. ant-t. arg-n. *arn.* ars. bar. bell. berb. borax. *bry. calc.* cann-i. *caps. carb-an.* caus. cham. cic. clem. cocc. con. cupr. dig. *graph.* hyos. *ign. k-ca.* k-iod. *lach.* led. lyc. merc. nat-c. *nat-m.* nit-ac. *nx-v. op. petr. phos.* ph-ac. plat. *puls.* rhus. *sabad. samb.* sep. *sil.* spong. *stram. sulph.* sul-ac. therid. verat.
— complaints from fright: ACON. arn. bell. bry. caus. cham. coff. cupr. gels. hyos. IGN. lach. lyc. nat-m. nx-v. OP. phos. plat. *puls.* samb. secale. stram. verat. znc.
— in dark: carb-an.
— at a noise: borax. cann-s. chel. hipp.
— — least noise: nx-v.
— — every sound: calc.
— nocturnal emission, after: aloe.
— in **sleep**: hepar. ign. merc. puls. rheum. stann. tabac.
— — starting from sleep in a fright: nx-v. tarent.
— at **trifles**: am-c. am-m. ang. ant-t. arn. calend. hyper. *k-ca.* k-iod. lach. merc. mezer. *nit-ac.* nx-v. phos. rhus. sep. sumb.
— — — day before menses: calc.
— on **waking**: eupion. lach. led. lyc. nit-ac. nx-v. sul-iod. verat. znc.
— awakens terrified, knows no one, screams, clings to those near: *stram.*
— awakens in a fright from least noise: nx-v.
— **wakening** one from sleep: euphr. sep.
— — — from a dream: abrot. chin. graph. mag-m. tarent.
— **weeping**, amel.: phos.
**Frivolous**: *arn.* bar. merc. par. spong.
**Frown**, disposed to: hell. lyc. stram.
**Fun**, full of: *cann-i.* nx-m. tarent.
— See also Mirth, etc.
**Fury**. See Rage. [etc.
**Gayety**. See Cheerfulness, Vivacity,
**Gentleness**: cyc. iod. nx-v.
**Gestures**, of usual avocations: stram.
— **convulsive**: alcoh. apis. bell. cann-s. plb.
— — at night, of drinking: bell.
— **furious**: cann-i. sep.
— **hands**, of the, as if brushing the face or as if brushing something away: hyos.
— — clapping of the: bell. cic. verat.
— — **grasping** or reaching at something: calc-p. cham. cina. hyos. *lyc.* mosch. *ph-ac. stram.* sulph. *znc.*
— — — at sides of the bed: nx-v.
— — — at bystanders: phos.
— — — at mother: *borax.*
— — — at genitals, during spasms: stram.
— — — quickly: *stram.*
— — **motions**, involuntary, of hands: ars. bell. cann-i. cic. coca. hyos. merc. mosch. nat-m. puls. sil. stram. sulph. verat.
— — — to the face: strych.
— — — folding hands: puls.
— — — to the head: plb. *stram.* verat.
— — — as if knitting: tarent.
— — — lifting up of hands: ars.
— — — rubbing together: cann-i.
— — — throwing about: atrop. bry. carb-an. mosch. nat-c. phos. sil. stram.
— — — — over head: hydr-ac. stram.
— — — waving in the air: stram.
— — — wild: acon.
— — — winding a ball, as if: agar. stram.
— **intoxicated**, gestures as if: hyos.
— **picks** at bed-clothes: *arn. ars.* atrop. bell. cham. chin. cocc. *colch.* con. dulc. hell. hepar. *hyos.* iod. *lyc.* mur-ac. *op.* phos. ph-ac. *psor.* rhus. sol-n. *stram.* sulph. *znc.* znc-m.
— — — at nose or lips: *arum-t.* con. hell.
— **plays** with his fingers: *hyos.*
— **pouring** from hand to hand, as if: bell.
— **pulls** hair of bystanders: bell.
— **ridiculous** or foolish: *bell.* cic. croc. cupr. *hyos.* ign. lach. merc. *mosch.* nx-m. op. *sep. stram.* verat.
— **violent**: agar. bell. camph. hyos.
— **wringing** of hands: phos. puls. sulph.
**Giggling**: cann-i.
— See also Laughing.
**Gloomy**. See Melancholy, Sad, etc.
**Godless**, want of religious feeling: anac. coloc.
**Good-humor**. See Cheerful.
**Gossiping**: hyos. stram. verat.
**Gravity**: cann-s. euph. grat. led. nx-m. sul-ac.
— at the ludicrous: anac.
**Greediness**: secale. sep. znc.

— acids, for: secale.
— constant: merc.
— in eating: agar.
— of mind: puls.
**Grief**: acet-ac. acon. agar. alum. am-c. am-m. ars. aur. bar. calc. carb-an. caus. coloc. cyc. *graph.* hyos. *ign. lach.* lact. *lyc.* nat-m. nx-v. op. ph-ac. puls. sep. *staph.* sul-ac. verat.
— Compare with Anxiety, Weeping, etc.
— about his condition: staph.
— for the future: nat-c. nat-m.
— undemonstrative: cyc. *ign.*
— and **sorrow**, bad effects from: ars. cocc. *coloc.* cyc. graph. *hyos.* IGN. lyc. nx-v. *ph-ac. puls.* STAPH. *verat.*
**Groaning**: *acon.* alum. am-c. ang. ant-t. ars. bar. *bell. bry.* calc. carb-an. cham. chin. cina. cocc. colch. *coff.* cupr. dig. gran. graph. hell. *ign.* ipec. k-ca. lach. mag-c. merc. mur-ac. naja. nat-c. nit-ac. *nx-v.* phos. plb. puls. rhus. sars. squil. stram. *verat.*
— See also Moaning.
— alternating with laughter, song: bell. stram.
— during sleep: alum. bry. calad. cham. clem. cocc-c. graph. ign. lach. mur-ac. plect. sep. *sulph.*
— when waking: cina.
**Growling** like a dog: alum. *bell.* hell. lyc.
— — See Howling.
**Grumbling**: alum. arn. bism. bry. calend. canth. cham. chin. phos.
**Grunting**: bell. hell. ign. puls.
— during sleep: ign.
**Hallucinations**. See Delusions.
**Hanging**, sees persons: ars.
**Happy**. See Cheerful.
**Hard-hearted**: anac. croc.
**Hastiness**: *ambr.* ars. aur. bar. bell. bry. cact. calc. cann-s. carb-an. con. dig. dulc. grat. hepar. k-ca. lach. laur. men. merc. nat-c. nat-m. nx-v. ox-ac. puls. sep. stram. *sulph.* sul-ac. viol-tr.
— Compare with Hurry.
— involuntary when taking anything: sulph.
— in mental work: ambr.
— with chill: cann-s.
— in talking: bell. hepar. ph-ac.
— in temper: naja. plb. sul-ac.
**Hatred**: agar. aloe. am-c. anac. aur. calc. cic. lach. led. mang. *nat-m.* nit-ac. phos. stann. sulph.
— of absent persons, better on seeing them: fluor-ac.
— of persons who have offended: *aur.* mang. *nat-m.* nit-ac. sulph.
— has bitter feelings for slight offenses: ang.
**Haughty**: alum. anac. arn. aur. cann-i. caus. chin. cic. cupr. dulc. ferr. guai. hyos. ign. ipec. lach. *lyc.* merc. nx-v. par. phos. *plat. stram.* stront. *verat.*
**Headstrong**. See Obstinate.
**Heedlessness**. See Carelessness.
**Helplessness**, feeling of: hell. k-bro. phos. taxus. stram.
— after vomiting: hell.
**Hesitation**: aur. bar. chin. *graph.* hyos. mezer. mur-ac. nx-v. *puls.* seneg. sil. sulph. thu.
— Compare Irresolute.
— in action and ideas: tarent.
— evening, on account of obstacles: ferr-p.
**Hide**, desire to: bell. lach. stram. tarent.
— — on account of fear: ars.
**High-spirited**: hydr. hyos. op. spig. spong. verat. verb.
**Hilarity**. See Mirth.
**Home**, desires to go: bell. bry. calc-p. *lach.* verat.
— See under Delirium.
**Home-sickness**: *aur.* bell. calc-p. *caps. carb-an.* caus. clem. eup-pur. hell. ign. mag-c. mag-m. merc. nit-ac. *petr.* ph-ac. plan. puls. senec. staph. verat.
— with red cheeks: *caps.*
**Hopeful**: acon. ferr-m. hydr.
— alternating with sadness: raph.
**Hopeless**. See Despondent.
**Horror**, evening: calc. carb-an. nat-m. phos. plat.
— worse in dark: cann-i.
— Compare with Anxiety, Fear, etc.
**Howling**: *acet-ac. acon.* alum. arn. ars. *aur.* bell. brom. calad. camph. caps. cham. cic. coff. cup-ac. ign. ipec. lyc. nx-v. op. stann. stram. tarent. *verat.* viol-tr.
— about future troubles: lyc.
— evening, worse in: VERAT.
— night, at: stram. *verat.*
— during heat: tilia.
**Humor**, agreeable: abrot. ant-t. croc. ign. lach. men. plat. sul-ac.
— **changeable**, variable, etc.: *acon.* agn. aloe. *alum.* ambr. anac. ant-t. arg. arn. ars. asaf. asar. aur. *bell.* bism. bov. borax. bry. cann-s. caps.

carb-an. caus. chin. cocc. con. *croc.* cupr. cyc. dig. dros. *ferr.* gels. graph. hyos. *ign.* iod. k-ca. lach. lachn. lyc. men. merc. mezer. nat-c. nat-m. nit-ac. nx-m. op. petr. phel. phos. *plat. puls.* ran-b. sabad. sars. seneg. sep. spig. spong. staph. *stram.* sulph. *sul-ac.* valer. verat. verb. *znc.*
— — for the various alternations of Humor see special names, as Anger, Laughter, etc.
— — in the evening: aur. croc.
— — after dinner: aloe.
**Humorous.** See Jokes.
**Hurry**: acon. ambr. ars. aur. benz-ac. calc. camp. cann-i. caps. carb-an. carb-v. cocc. con. graph. hyos. ign. k-ca. lach. laur. lil-t. merc. nat-c. nat-m. op. phos. ph-ac. puls. sep. stram. sulph. sul-ac. thu. viol-tr.
— Compare with Hastiness.
— as if by duties: *lil-t.*
— in eating: lach. pip-m.
— in mental work: ambr. aur. ign. k-ca. op.
•— in movements: acon. bell. camph. cann-i. coca. coff. con. gins. *hyos.* men. merc. merl. stram. *sulph.* viol-tr.
— — cannot do things fast enough: aur.
— in occupation: acon. aur. camph. k-ca. op. puls. sep. sul-ac. viol-tr.
— — desires to do several things at once: plan.
— in pace: stram. sulph.
— in speech: acon. ign. merc. ph-ac.
— in writing: ptelea.
**Hydrophobia**: acet-ac. arg-n. *bell. canth.* hydrph. *hyos. lach.* ran-sc. *stram.* verat.
**Hypochondriacal** humor. See Melancholy, Sad, etc.
**Hypocrisy**: phos.
**Hysterical**: acon. anac. *asaf.* aur. bell. bry. calc. camph. cann-s. caul. caus. cham. chin-s. cic. cocc. con. croc. elaps. ferr. grat. hyos. ign. ipec. k-ca. lact. lil-t. mag-c. mag-m. merc. mill. *mosch.* nat-m. nit-ac. nx-m. nx-v. op. phos. plat. plb. puls. *raph.* sep. sil. stann. staph. stram. sulph. TARENT. therid. valer. viol-od.
— lascivious: tarent.
— first day of menses: raph.
— moaning aggr., sighing amel.: tarent.
**Ideas.** See Thoughts.

**Idiocy**: absin. æth. agar. ant-c. bar-m. hyos. merc. nx-m. plb. tabac.
— idiotic actions: ant-c.
**Idleness.** See Indolence.
**Ill-humor** (crossness, irritability, etc.): abrot. acet-ac. acon. æsc-h. æth. agar. aloe. alum. am-c. am-m. *anac.* ang. ANT-C. ant-t. apis. *arg.* arn. *ars.* arum-t. asaf. asar. *aur.* bar. *bell.* berb. *bism.* borax. bov. brom. BRY. bufo. cact. cadm. calc. *calc-p.* calend. camph. cann-s. canth. *caps.* carb-an. carb-v. *caus.* CHAM. chel. chin. chin-s. cic. *cina.* cinnb. clem. cocc. cocc-c. colch. coloc. *con.* cop. coral. corn. *creos.* croc. croton. cupr. *cyc.* daph. dig. dios. dros. dulc. elaps. euphr. eupion. evon. ferr. fluor-ac. gels. gran. graph. grat. guai. ham. hell. helon. hepar. hipp. hydr. hydr-ac. hydrph. ign. *indg.* iod. ipec. iris. jatr. k-bi. k-ca. k-iod. kalm. lach. lachn. lact. laur. led. lil-t. *lyc.* mag-c. mag-m. manc. mang. marum. men. meph. merc. merc-i-r. merl. mezer. mosch. mur-ac. naja. *nat-c.* nat-m. nat-s. nicc. nitr. nit-ac. nx-m. *nx-v.* ol-an. *olnd.* op. osm. *petr.* phel. *phos.* ph-ac. pic-ac. plan. *plat.* plect. prun. psor. PULS. ran-b. ran-sc. ratan. rheum. rhod. RHUS. rhus-v. ruta. sabad. sabin. *samb.* sang. sars. selen. SEP. *sil.* spig. spong. squil. stann. *staph.* stram. stront. *sulph. sul-ac.* tabac. *tarent.* thea. tilia. *thu.* tong. tril. valer. verat. verb. viol-tr. vinc. znc.
— Compare with Fretful.
— **alternating** with cheerfulness: ant-t. aur. chin. cyc. lyc. merc. nat-c. nat-m. plat. spig.
— — — hypochondriacal mood during day, merry in evening: sulph. viol-tr.
— — — indifference: bell. carb-an.
— — — melancholy: znc.
— — — tenderness: plat.
— — — weeping: bell.
— toward children: k-iod.
— nervous irritability: ars-iod. asar. carb-an. caus. lach. lyc. plb.
**Conditions** of ill-humor.
— **morning**: am-c. am-m. ant-t. bov. calad. calc. cast. cham. chin. cocc. con. creos. graph. grat. hipp. mag-c. *mang.* merc-i-r. *nat-m.* nat-p. nicc. phos. plat. sars. seneg. sep. sil. *staph.* tarax. verat.

— — on waking: agar. arg-n. ars. bell. bov. bry. carb-an. coca. gamb. iris. k-ca. lil-t. lyc. mag-m. merc-iod. mezer. nat-m. nat-s. nit-ac. nx-v. petr. ph-ac. plat. plb. puls. rhus. sulph. sul-ac. thu.
— — after rising: calc. canth. coff. hepar. mag-m. nat-s. sulph.
— **afternoon**: æth. alum. ant-t. borax. bov. cast. chel. con. cyc. elaps. hydr-ac. mang. merc-c. nat-m. op. ox-ac. ruta. sang. sars. znc.
— **evening**: æsc-h. aloe. am-c. am-m. ant-c. ant-t. bar. bov. calc. canth. cast. cyc. dios. hydrph. ign. indg. k-ca. kalm. lyc. lil-t. lyc. mag-c. mag-m. mur-ac. nicc. nx-j. ox-ac. pallad. phos. puls. ran-b. sil. spig. sulph. znc.
— — amel. in: aloe. am-c. bism. mag-c. nat-m. nicc. verb. znc.
— **night**: camph. phos. *rhus.*
— — on waking: lyc.
— **air**, in open: æth. plat. rhus.
— — amel.: coff. mag-c. rhus. stann.
— **aroused**, when: op. nx-m.
— **causeless**: aloe. chel. cyc. merc-i-r. *nat-m.*
— **children**, in: ant-c. ant-t. ars. borax. graph. lyc. puls. sep. znc.
— **chill**, during: calend. nat-m.
— — See also under Fever.
— **coffee**, after: calc-p.
— **coition**, after: nat-m. selen.
— — during an emission: clem.
— **contradiction**, from: grat. *ign.* sil. tarent.
— **cough**, before: asaf. bell.
— **diarrhœa**, after: guai.
— **digestion**, during: iod.
— **dinner**, before: phos.
— — during: marum.
— — after: cocc-c. mill. marum. nat-c. tilia. [op.
— **disturbance**, cause by any: graph.
— **dreams**, from: op.
— **eating**, after: am-c. ars. bov. bry. carb-v. cham. con. graph. iod. k-ca. k-iod. marum. merc. merc-sul. nat-c. nat-m. nx-v. puls.
— — amel.: am-c. nat-s.
— **excited**, when: chin.
— **fever**, after: am-c. hipp.
— — during the hot stage (of intermittent): acon. *ars. cham.* lach. mosch. NAT-C. psor. *rheum.* staph.
— — during the chill: arn. *ars.* CALC. calen. CAPS. *caus. cham.* CON. creos. hepar. ign. LYC. mezer. nat-m. nit-ac. petr. phos. PLAT. *puls.* RHEUM. rhus. sil. *spig. sulph.*
— — during the sweat: ang. bry. *calc.* calc-p. *cham.* clem. hepar. mag-c. merc. RHEUM. sep. *sulph.*
— **fly** on the wall, by: sars.
— **gastric** troubles, with: znc.
— **headache**, during: acon. æth. *am-c.* am-m. anac. bell. bov. bry. calc-p. chin-s. coca. con. creos. cyc. dulc. graph. helon. *hipp.* iod. k-ca. lach. lachn. laur. mag-m. mang. merc. nat-m. *nicc.* op. pallad. plat. *phos.* sil. spong. stann. thu. vip. znc.
— **menses**, before: cham. creos. lyc. mag-m. nat-m. nx-v. znc.
— — during: berb. bry. cast. caus. *cham.* cimic. cina. creos. eupion. ferr. k-ca. lyc. mag-c. mag-m. mag-s. nat-m. *nx-v.* plat. sep. sulph. tarent. znc.
— — after: berb. bufo. ferr. nat-m.
— — during an intermission of: eupion.
— **music**, during: caus. *mang.*
— **nausea**, during: phos.
— **noise**, from: pip-m. plect.
— **pain**, after: *cham.* croton. hepar.
— — in abdomen: aloe. am-c. berb. phos. tarent.
— **perspiration**, during: ang. calc-p. clem. mag-c. sep.
— — See also under Fever.
— **provoked**, when: verat.
— **questioned**, when: nat-m. ph-ac.
— **reading**, when: nat-c.
— **room**, house, in: ign. *rhus.*
— — amel.: æth.
— **sitting**, when: mang. nat-m.
— **sleep**, after: anac. bell. bry. caus. cham. clem. jatr. mezer.
— — See also Morning, on waking.
— **sleepiness**, during: calc. calend. canth. lachn. rhus-r. spong.
— **words**, soothing or friendly persuasion, worse from: bell. calc. *calc-p.* chin. *ign.* k-ca. merc. *nat-m.* nit-ac. nx-v. plat. *sep. sil.*
— **spoken** to, when: ars. cham. gels. nat-m. nat-s. nx-v. rhus.
— **stool**, before: aloe. *borax.* calc.
— — after: nat-c. nit-ac.
— **talking**, when: ambr. cham. mang. marum. nicc. staph. sul-ac. znc.
— **toothache**, during: merc-i-r.
— **trembling**, with: ambr. arg-n. aur. chel. cop. daph. nit-ac. phos. sep.

— **trifles**, about: ang. *bell.* carb-v. caus. *cham.* chel. con. *cyc.* hepar. lyc. meph.
— — See also Causeless.
— **waking**, on: ant-t. bell. cham. chel. clem. *cyc.* iris. nat-m. tarent.
— — See also under Morning.
— **walking** in open air, amel.: mag-c. *rhus.*
— **weakness**, with: apis. arg-n. calc. caus. crotal. grat. hipp. merl. phos. ph-ac. spong.
— **weather**, in rainy or cloudy: aloe. am-c.

**Illness**, apprehension of: acon. alum. am-c. ars. borax. bry. calad. *calc.* creos. ign. k-ca. *lach.* merc. nat-c. nat-m. *nx-v.* phos. ph-ac. *puls.* sep. sulph.
— sense of, in mind and body: merc.

**Illusion** of fancy: acon. æth. agar. ambr. anac. ang. ant-c. arn. *ars.* aur. bar. *bell.* berb. bism. bry. calc. camph. cann-s. canth. carb-an. carb-v. caus. cham. chin. chin-s. cic. cina. *cocc.* coff. colch. coloc. con. croc. cupr. dig. dros. dulc. euphr. graph. hell. hepar. *hyos. ign.* indg. iod. k-ca. lach. led. lyc. mag-m. mag-s. merc. nat-c. nit-ac. nx-m. nx-v. *op.* par. phos. *ph-ac.* plat. plb. puls. rheum. rhod. *rhus. sabad.* samb. secale. sep. sil. spong. stann. staph. *stram. sulph.* thu. valer. verat. verb. viol-od.
— in **discerning** objects: calc. cann-s. hyos. nx-v. plat. sulph.
— of **feeling**: *anac.* ant-t. bell. canth. ign. iod. mag-m. nx-v. op. petr. phos. plat. rhus. sabad. *stram.* sulph. valer.

**Illusions.** See Delusions.

**Imaginations.** See Delusions.

**Imbecility**: acon. agar. alum. *anac.* ant-c. arg-n. ars. asar. aur. bar. bell. bov. calc. cann-s. caps. cham. cocc. con. croc. cupr. cyc. dulc. hell. *hyos.* ign. k-bi. *lach.* laur. lyc. merc. mezer. mosch. mur-ac. *nat-c* nat-m. nx-m. nx-v. olnd. *op.* par. petr. *ph-ac.* plb. puls. ran-b. rheum. rhus. ruta. sabad. secale. selen. seneg. sep. sil. spong. stann. staph. stram. sul-ac. verat.

**Impatience**: acon. ambr. *ars.* aur. bar. bufo. calc. carb-v. *cham.* chin. coloc. dros. *dulc.* gels. hell. hepar. hyos. *ign. ipec.* k-bi. *lach.* lil-t. lyc. merc. nat-c. nat-m. nicc. nit-ac. nx-v. op. pallad. ph-ac. plan. *plat.* psor. puls. rheum. rhus. sars. sep. *sil.* spong. sulph. sul-ac. tarent. taxus. znc.
— during dinner: sulph.
— during headache: pallad.
— from itching: osm.
— — pain: hura.
— — playing of children: anac.
— while reading: nat-c.
— when sitting: sep.
— after supper: nit-ac.
— about trifles: merc. *sul-ac.*
— before urinating: sulph.
— when walking: lyc.

**Imperious.** See Haughty.

**Impertinence**: graph.

**Impetuous**: acon. anac. *bry. carb-v.* cham. croc. ferr-p. hepar. k-iod. laur. led. nat-c. nat-m. nit-ac. *nx-v.* olnd. phos. rheum. sep. stront. *sulph. znc.*
— Compare with Hastiness, Hurry, Impatience, etc.

**Importance**, assumption of: cupr. ferr. *hyos.* lyc. *stram.* verat.

**Impudence**: graph. phos.
— See Shameless.

**Impulsive**: arg-n. ars. *cic.* gins. merc.

**Inattention.** See under Attention, Memory, etc.

**Inciting** others: hyos.

**Inconsolable**: *acon.* ambr. *ars. cham.* chin. coff. nat-c. *nx-v. spong.* stram. sulph. verat.
— over fancied misfortune: *verat.*

**Inconstancy**: asaf. bism. canth. coff. dros. ign. led. op. sil. spig. thu. znc.
— Compare with Irresolute.

**Indifference**, apathy, etc.: agar. agn. alum. ambr. am-m. *anac.* apis. arg-n. arn. *ars.* asaf. asar. bell. berb. bism. bov. brom. calc. calc-p. camph. cann-i. caps. carb-an. carb-v. caus. cham. chel. chin. cic. cimic. cina. clem. cocc. con. croc. crotal. cupr. cyc. dig. elaps. euphr. ferr. fluor-ac. gels. glon. graph. gymn. hell. hyos. ign. iod. ipec. jatr. k-bi. k-ca. lac-can. lach. laur. lyc. manc. men. merc. mezer. mur-ac. naja. nat-c. *nat-m.* nit-ac. *nx-m.* nx-v. olnd. *op. phos. ph-ac.* phyt. pic-ac. plat. prun. *puls.* raph. rheum. rhod. rhus. ruta. sabad. sabin. *secale.* selen. seneg. *sep.* sil. squil. stann. staph. stram. sulph. tarent. thu. *verat.* verb. viol-tr. znc. ziz.
— **alternating** with anxiety and restlessness: nat-m.
— — — cheerfulness: tarent.
— — — ill-humor: asaf. bell. carb-an.
— — — vexation: cham.

— — — weeping: phos.
— does not complain: hyos. op.
— — — — unless questioned, then says nothing of his condition: colch.
— has no desire, no action of the will: hell.
— with insensibility, with pale face: chin.
— — — rubs forehead: verat.
— to **agreeable** things: ambr. anac. cina. corn. op. rhod. staph. stram.
— **business** affairs: arg-n. arn. fluor-ac. sep. *stram.*
— to **everything**: acet-ac. agar. ailan. anac. arn. bell. bov. canth. caps. carb-v. croc. dig. hydr. ign. lept. merl. mezer. nx-m. phos. secale. staph. sulph. ziz.
— to **external** things: agn. cann-i. cham. cic. lyc. merl. op. rumx. stann. staph. tarent. verat.
— to **important** things: fluor-ac.
— to **irritating**, disagreeable things: ambr. anac. borax. cina. coff. op. rhod.
— to **loved** ones: merc. sep.
— to **relations**: fluor-ac. hell. hepar. phos. plat. sep.
— — — her children: phos. sep.
— toward others: sulph.
— **well**, says he is: arn. ars.
— **morning** on waking: phyt.
— **afternoon**: con.
— **evening**: dig. k-clc.
— **air**, in open: con. plat.
— **onanism**, after: staph.
— **solitude**, by: bov.
— **society**, when in: plat.
— — amel.: bov.
— **walking**, when: con.

**Indignation**: ambr. *ars.* calc-p. chin. coloc. nat-c. staph.
— bad effects following: *coloc.* ipec. nx-v. plat. staph.
— discomfort from general: op.
— dreams, at unpleasant: calc-p.

**Indiscretion**: acon. bry. calad. camph. caps. hyos. ign. laur. men. *nx-v.* puls. stram.

**Indolence**: abrot. acon. alum. am-c. am-m. anac. ant-c ant-t. arg. arn. ars. asaf. asar. aur. bar. bell. borax. bry. calc. camph. cann-s. canth. carb-an. carb-v. *caps.* caus. chel. chin. chin-s. cinnb. cocc. con. croc. crotal. dig. euphr. graph. guai. hell. hyos. ign. iod. ipec. k-ca. *lach.* laur. lyc. mag-c. mag-m. marum. meph. mezer. mur-ac. nat-c. nat-m. nit-ac. nx-j. *nx-v.* olnd. op. petr. phos. ph-ac. plat. plb. puls. ran-sc. rheum. ruta. sabin. secale. sep. spig. spong. squil. stront. sulph. verb. znc.

**Conditions** of indolence.
— **morning**: all-c. aloe. alum. anac. ant-t. carb-an. chel. clem. hepar. hipp. indg. mag-c. nat-c. *nat-m.* nat-s. nitr. nx-v. ox-ac. pallad. phyt. plat. ran-sc. rhus. rumx. sabin. squil. sulph. tarax. verb.
— — on rising: dig. nat-c. op. verb.
— — on waking: chin-s.
— **afternoon**: aloe. *borax.* bufo. chel. hyos. lyc. mag-s. nat-m. petr. sep. sil. viol-tr.
— **evening**: agar. calc-p. cann-s. carb-v. coca. dios. ferr-iod. mag-c. pallad. plb. ran-sc. spig. sulph. viol-tr.
— — amel. in: aloe. bism. clem.
— **air**, in open: arn.
— — amel.: calc.
— **breakfast**, after: nat-s.
— **business**, when doing: nx-v.
— **chill**, during: camph.
— **emission**, after an: sep.
— **headache**, during: dulc.
— **meals**, after: agar. asar. ant-c. bar. bov. cann-i. *chin.* lach. mag-c. nx-v. *phos.* plb. thu. tong. znc.
— **sitting**, while: nat-c. nit-ac. ruta.
— **sleep**, after: mezer.
— **stool**, after: colch.
— **walking**, when: arn. chin-s. nit-ac. sabin.

**Industrious**: arn. aur. bell. bry. calc. caps. dig. hyos. *ign.* ipec. *lach.* mag-c. mosch. nat-c. plb. stann. sul-ac. verat.

**Inhumanity**: anac. op.

**Injure**, fears to be left alone lest he should do himself bodily harm: ars.
— feels as if she could easily injure herself: sep.
— satiety; must use self-control to prevent shooting himself: *nat-s.*
— See also under Suicide.

**Insanity**, madness: acon. æth. agar. all-c. am-c. anac. ant-c. ant-t. arg-n. arn. *ars.* aur. bar. *bell.* calc. camph. cann-s. canth. carb-an. caus. chin-s. cic. cocc. coloc. con. *croc.* crotal. cupr. dig. dulc. *hyos.* ign. k-ca. k-bi. k-ox. *lach.* led. *lyc.* merc. mezer. naja. nat-m. nx-m. *nx-v.* olnd. op. oxal-ac. par. phos. ph-ac. phys. plat. plb.

puls. rhod. rhus. sabad. secale. seneg. sep. sil. squil. *stram.* sulph. *tarent.* *verat.* verat-v. znc.
— Compare with Delirium, Mania, etc.
— **alternating** with stupor: op.
— — — mental symptoms: sabad.
— **disputing**: camph.
— **destructive**: verat.
— **funny**: croc. stram.
— **loquacious**: agar. lach. par. stram.
— **mild**: croc. verat.
— **proud**: hyos. stram. verat.
— **raving**: hyos. stram.
— **religious**: verat.
— **timorous**: bell.
**Conditions** of insanity.
— **abdomen**, with pain in: canth. cupr.
— **appetite**, with loss of: verat.
— **blood**, with discharge of: merc.
— **breathing**, with oppression of: merc.
— **chilliness**, with: calc.
— — coldness of skin: crotal.
— **cough**, with: bell. verat.
— **drunkards**, in: *ars.* bell. calc. carb-v. chin. coff. dig. hell. hepar. hyos. lach. merc. nat-c. *nx-v.* *op.* puls. *stram.* sulph.
— **emaciation**, with: sulph.
— **eruptions** suppressed, after: bell. stram. sulph.
— **face**, with heat of: verat.
— — with redness of: calc. op.
— — with paleness of: croc. merc. verat.
— — wild look: cupr.
— **feet**, with stamping of: verat.
— **fright** or anger, caused by: plat.
— **gesticulations**, with: bell.
— **hair**, pulls the: tarent.
— **headache**, with: croc. verat.
— **heat**, with: bell. verat.
— **larynx**, with pains in: canth.
— **legs**, with restless: tarent.
— **menses**, with profuse: sep.
— — with suppressed: puls.
— — during climacteric period: aster. lach. puls. sep. therid.
— **mouth**, with distortion of: ph-ac.
— **neuralgia**, with disappearance of: cimic.
— **ophthalmia**, with: croc. op.
— **parturient**, in the: bell.
— **perspiration**, with, following: cupr.
— **ptyalism**, with: verat.
— **pulse**, with quick: ars. crotal. cupr.
— **sight**, with obscuration of: croc.
— **sleep**, with: ph-ac.
— **trembling**, with: ars.
— **vomiting**, with: cup-ac.
— **wantonness**, with: hyos. stram. verat.
**Insensibility**, mental: cyc. hell. *hyos.* laur. op. phos. ph-ac. sabad. secale. stram.
— See also Unconsciousness.
**Insipid** behavior (dull, spiritless): acon. anac. carb-an. carb-v. nx-v. par. seneg.
**Intellect** predominates over feeling: viol-od.
— See also Mind, Thought, etc.
**Intolerance** of, open air: cocc. op.
— Compare with Loathing, Sensitiveness, etc.
— — **ailment**, of any: nx-v.
— — **annoyance**: ferr-p.
— — **bath**, of usual: clem. *sulph.*
— — **bed-covers**: led.
— — **contradiction**: aur. nx-v.
— — **drink** and food: ant-t.
— — **interruption**: cham. cocc.
— — **jar**, of the least: *bell.*
— — **looked** at, of being: *ant-cr.* ant-t. ars. cham. cina.
— — **music**: ambr. cham. sabad.
— — — instrumental: nat-c. nx-v. phos. sep.
— — — vocal: nx-v.
— — **noise**: acon. ambr. am-c. ars. bell. calc. chin. cocc. con. ign. iod. ipec. manc. nat-c. nit-ac. nx-v. phos. ph-ac. ptel. sep. sil. tanac. therid.
— — — of the slightest: acon. cinnb.
— — — of a hammer: manc.
— — — of sawing wood: manc.
— — — of steps: nit-ac. nx-v. sang.
— — — of water splashing: nit-ac.
— — — of the voice: nx-v. sil. znc.
— — **spoken** to, of being: ars. *cham.* gels. nat-m. nat-s. nx-v. rhus.
— — **uncovered**, of being: acon. æth.
— — **wind**, of: caus. *cham.* lach.
**Irascibility**. See Anger.
**Irresolute**: alum. ang. ars. asaf. bar. bism. bry. calc. cann-i. canth. cham. chin. coca. cocc. cupr. daph. ferr. graph. grat. hell. hyos. *ign.* iod. k-ca. lach. led. lyc. mag-m. mezer. nat-c. nat-m. nx-m. nx-v. op. pallad. *petr.* phos. plat. plb. *puls.* rhus-r. ruta. sil. spig. sulph. tarax. tarent. thu. znc.

— about trifles: bar.
— in acts: lyc. nat-c. nx-m.
— in ideas: nat-m. sulph.
— in projects: bar-ac. rhus-r.
— in making up his mind: bar-ac. graph. nat-m.
**Irksome**, everything is: ign. laur. lyc.
**Irrational**: bell. op. plb.
**Irritability**. See Fretful, Ill-humor, etc.
**Irritated** easily, is: anac. bar. bell. bry. croc. hepar. ign. ipec. nat m. *nx-v.* olnd. petr. ph-ac. sabad. squil. stram. tarax. verat.
— See also Fretful.
**Isolation**, feeling of. See Abandoned; Forsaken.
**Jealousy**: *apis.* camph. gal-ac. *hyos.* ign. lach. nx-v. op. ph-ac. puls. raph.
— as foolish as it is irresistible: lach.
**Jokes**, jesting, etc.: aloe. bell. bry. cann-i. caps. *cic. cocc.* glon. hyos. *ign.* ipec. k-iod. lach. lyc. merc. merl. nat-m. nx-v. plat. rhus-r. sars. secale. *stram.* sul-ac. tabac. *tarent*
— after indifference: men.
— — gravity: plat.
— **averse** to: *acon.* am-c. ang. apis. ars. borax. bov. carb-an. cocc. cyc. merc. nat-m. nx-v. puls, sabin. sil. spig. sulph. thu.
— **ridiculous** or foolish: bell. *cic.* hyos. *stram.* tanec. *verat.*
**Joy**, ailments from excessive: acon. caus. *coff.* croc. *op.* puls.
**Joyous**. See Cheerfulness.
**Kicks**: *bell. stram.* strych. tarent. verat-v.
— when carried: cham.
— in sleep: bell. cina.
— child is cross, kicks and scolds on waking: lyc.
**Killed**, desires to be: *ars.* bell.
— sudden impulse to kill for a slight offense: hepar. nx-v.
— See also Murder.
**Kind** words, friendly persuasion, etc., cause aggr.: *bell.* cact. calc. calc-p. chin. hell. IGN. k-ca. lyc. nat-m. nit-ac. nx-v. *plat.* SEP. SIL. staph.
**Kisses** every one: croc. verat.
— his companion's hands: agar.
— before menses: verat.
**Kleptomania**: absin. ars. bry. k-ca. lyc. nx-v. puls. sep. sulph.
**Kneeling** and praying, etc.: stram.
— unable to kneel down: tarent.

**Lamenting**, bemoaning, wailing, etc.: *acon.* alum. am-c. arn. *ars.* asaf. bell. bism. brom. bry. calc. camph. canth. cham. chin. cic. cina. cocc. *coff.* cupr. hell. hyos. ign. ipec. lach. lyc. merc. mosch. nat-c. nit-ac. nx-m. *nx-v.* op. phos. ph-ac. plb. puls. ran-b. rheum. rhus. secale. sil. sulph. tarent. verat.
— Compare with Weeping.
— during convulsions: ars.
— while asleep: arn. *cham.* cin. phos. sulph.
— over trifles: *coff.*
**Lasciviousness**. See Lewdness.
**Laughing**: acon. agar. aloe. alum. am-c. anac. apis. arn. asaf. aur. *bell.* calc. *cann-i.* cann-s. carb-v. cic. coff. con. coral. creos. *croc.* cupr. ferr. graph. hell. *hyos.* ign. k-bi. lach. lyc. merl. nat-m. *nx-m.* nx-v. op. phos. plat. plb. puls. ran-sc. sabad. sep. *stram.* sulph. tabac. tarax. tarent. verat. verb. znc. znc-s.
— **alternating** with frenzy: stram.
— — — groaning: bell. stram.
— — — sadness: canth. caus. nat-c. stram. znc.
— — — seriousness: nx-m. plat.
— — — vexation, ill-humor: croc. stram.
— — — violence: croc. stram.
— — — weeping: acon. alum. *aur.* borax. cann-s. caps. *coff.* graph. lyc. phos. *puls.* sep. stram. sulph. verat.
— — — child cries and laughs easily; while crying, it suddenly laughs heartily, and finally cries again: *coff.*
— — — whining, moaning: verat.
— **annoying**: bell.
— **aversion** to: ambr.
— **constant**: cann-i. verat.
— **convulsive**: bell. calc. cupr. ether.
— **delirious**: stram.
— **easily**: ars. *coff.*
— **feats**, at his own: iris. stram.
— **forced**: hyos.
— **immoderately**: cann-i. ferr.
— **involuntarily**: agar. bell. *cann-i.* phos.
— — after eating: puls.
— **loudly**: bell. cann-s. croc. hyos. op.
— **misfortune**, at: apis.
— **mocking**: tarent.
— **nervous**: hura.
— **paroxysmal**: stram.
— at reprimands, etc.: graph.
— **silly**: bell. crot-c. ether. hyos. stram.

— **spasmodic**: acon. alum. am-c. anac. asaf. *aur.* bell. *calc.* cic. *con.* croc. cupr. ether. *ign.* lyc. nat-m. nx-m. phos. plat. secale. sil. stram. valer. verat. znc.
**Conditions** of laughing.
— **air**, in open: nx-m.
— **morning**: graph. hura. plat.
— **night**: cic. creos. sil.
— **anxiety**, during: lyc.
— **sad**, when: phos.
— followed by sadness: plat.
— **serious** thoughts, when occupied by: *anac.* lyc. nat-m. sulph.
— **sleep**, during: alum. caus. *hyos.* lyc. ph-ac.
— **speaking**, when: bell.
**Lewdness**, lasciviousness, lustful, etc.: bov. calc. coc-c. fl-ac. graph. hyos. ign. lach. lyc. nat-m. op. sil. *stram.* tarent.
— See also Shameless and under Mania, Sexual, etc.
— lewd talk: plat.
— — fancies: dig. sang.
**Listless**. See Apathy.
**Lively**. See Merry.
**Loathing**, general: acon. ang. ant-t. arg-n. arn. asar. bell. benz-ac. bufo. calc. canth. cham. chel. hyos. jatr. k-bi. k-ca. merc. phel. plat. *puls.* ratan. secale. seneg. stram. sumb. tarent. thea.
— — Compare with Intolerance.
— — morning on waking: phyt.
— — evening: alum. hepar. raph.
— — eruption, before: cop.
— — pain during: aloe.
— — smoking, when: sep.
— **against** bananas: elaps.
— — **bread**: elaps. tarent.
— — **butter**: *chin.*
— — **chocolate**: tarent.
— — **coffee**: phys.
— — **drinks**: phys. secale.
— — **everything**: nx-v. therid. thu.
— — **food**: all-c. anac. cham. corn. dios. eup-per. hell. k-ca. mag-c. merc-iod. nx-v.
— — **life**: agn. alum. ambr. am-c. ant-cr. ant-t. ars. AUR. aur-m. bell. bov. carb-an. caus. chin. creos. dros. hepar. hyos. k-bi. lach. led. lyc. merc. mezer. nat-c. nat-m. nat-s. nit-ac. *nx-v.* op. phos. plat. plb. *puls.* rhus. ruta. secale. sep. sil. spig. spong. staph. stram. sulph. sul-ac. thu.
— — — must restrain herself to prevent doing herself injury: nat-s.
— — — evening: *aur.*
— — **liquors**: ang.
— — **meats**: agar. crot-c. elaps. tarent.
— — **speaking**: anac. dios.
— — **tea**: thea.
— — **veal**: phel.
— — **work**: arn.
**Locality**, errors of: bry. *glon.* hura. lach. nx-m. par. petr. sulph. valer. verat.
— — loses way in well-known places: *glon.* nx-m. petr.
**Looked** at, children cannot bear to be: *ant-cr.* ant-t. ars. cham. cina.
**Loneliness**, a feeling of: calc. cann-i. k-ca. mag-m. nat-c.
— See also Forsaken.
**Longing** for things which are rejected when offered: ars. bell. bry. *cham.* cina. dulc. hepar. puls. rheum. staph.
— — — but does not know what: china.
— for good opinion of others: pallad.
— for repose, tranquillity: nx-v.
— for sunshine, light, and society: stram.
— See also name of thing desired.
**Lie**, never speaks the truth; does not know what she is saying: verat.
— believes all she says is a: lac-can.
**Loquacity**: abrot. acon. æth. agar. agn. aloe. ambr. arg. ars. aur. bapt. bell. borax. bov. calc. cann-i. canth. caus. cimic. cocc. coc-c. coff. croc. cupr. dulc. ether. eugen. gamb. gels. glon. grat. *hyos.* iod. k-iod. *lach.* lachn. lil-t. mag-c. marum. meph. nat-c. nat-m. nicc. nx-m. op. par. psor. rhus. *selen.* stann. staph. *stram.* tabac. tarax. tarent. thea. therid. thu. verat. viol-od. znc.
— **alternating** with laughing: bell.
— **changing** quickly from one subject to another: agar. cimic. *lach.*
— **evening**: lach. viol-tr.
— **excited**, when: selen.
— **measles**, with: viol-od.
— **menses**, during: stram.
— **sleep**, during: ambr. ign. op.
— See also Speech.
**Love**, ailments from disappointed: calc-p. cimic. coff. *hyos.* *ign.* lach. ph-ac. staph.
— love-sick: *ant-cr.* tilia.
— with jealousy, anger, and incoherent talk: hyos.

— with silent grief: *ign.*
**Low-spirits.** See Sadness.
**Ludicrous**, things seem: cann-s. nat-m. *nx-m.* stram.
**Magnetized**, desires to be: calc. phos.
**Malice**: *acon.* agar. am-c. am-m. *anac.* arn. ars. aur. bar. *bell.* borax. cann-s. canth. caps. carb-an. caus. chin. cic. cocc. coloc. con. croc. cupr. guai. hepar. *hyos.* ign. *lach.* led. lyc. mang. merc. mosch. nat-c. nat-m. nit-ac. *nx-v.* op. par. petr. phos. plat. secale. squil. *stram.* stront. verat. znc.
**Mania**, madness: acon. æth. ailan. alcoh. ant-cr. ars. bell. cact. camph. cann-i. canth. chin-s. coca. coff. colch. con. crotal. cupr. hell. hyos. indg. k-iod. led. lyc. merc. merc-c. nx-m. œna. op. phos. plb. raph. rhod. sabad. secale. stram. tarent. tereb. verat.
— Compare with Delirium, Insanity, etc.
— amel. by washing head in cold water: sabad.
— **anxiousness**, with: bell.
— **bites**: bell. bufo. hyos. secale. stram.
— **disgust** of life, with: bell.
— **capricious**: raph.
— **crawls** on floor, spits often, hides, laughs, or is angry, with spasms: lach.
— **declares** she will go crazy: cimic.
— **furious**: canth. hyos. op. stram.
— **gesticulates**: hyos. nx-m. stram.
— **laughing** and gayety, with: acon. bell. caus. croc.
— — with groaning and laughing: stram.
— — metrorrhagia, alternating with: crot-c.
— **neuralgia**, after sudden disappearance of: cimic.
— **prays**: stram. verat.
— **quarrelsome**: cupr. hyos. lyc.
— **runs** away: bell. cupr. dig. nx-v. verat.
— — about: bell. con. hell. stram. sulph. verat.
— — — naked: bell. hyos.
— **sexual**: apis.
— **sings**: bell. cann-i. cocc. cupr. hyos. stram. verat.
— **study**, from over: *lach.*
— **talkative**: agar. *lach.* par. *stram.*
— **tearful**: acon. cann-s.
**Mania-a-potu**: acon. agar. ant-cr. arn. *ars.* bell. *calc.* cann-i. carb-v. chin. cimic. coff. dig. hell. hyos. ign. lach. led. lyc. merc. nat-c. nat-m. nx-m. *nx-v.* op. puls. ran-b. rhod. rhus. ruta. selen. sep. sil. spig. stram. sulph. sul-ac. verat. znc.
**Marriage**, the idea of, seemed unendurable: pic-ac.
**Meditation**: am-c. cann-s. canth. chin. cic. clem. cocc. cyc. hell. hyos. ign. lach. mezer. nat-c. phel. phos. plb. ran-b. rhus. sabad. sep. spig. staph. sulph. thu.
**Melancholy**, Gloomy, etc.: abrot. acon. æsc-h. all-c. alum. ambr. am-c. am-m. anac. ant-cr. arg-n. *ars.* asar. *aur. bell.* berb. bolet. bov. cact. *calc.* calc-p. canth. caps. carb-an. *caus.* chel. chin. chin-s. clem. cocc. con. creos. croc. crotal. cupr. cyc. dig. dros. euph. euphr. ferr. gamb. *graph. hell.* hepar. *hyos.* ign. indg. iod. iris. k-bi. k-ca. lac-can. *lach.* lact. laur. lil-t. *lyc.* merc. nat-c. *nat-m.* nit-ac. nitr. nx-m. nx-v. op. petr. phos. ph-ac. plan. plat. *plb. puls.* ran-sc. *rhus.* ruta. sabad. sars. secale. selen. *seneg. sep.* sil. spig. stann. staph. *stram. sulph.* sul-ac. tabac. tilia. verat. viol-od. znc. zing.
— Compare with Despair, Despondency, Grief, Sadness, etc.
— **alternating** with cheerfulness: asar. chin. ferr. znc.
— — — ill-humor: znc.
— **religious**: graph. psor. sulph.
— — See Fear of salvation, Religious, etc.

**Conditions** of Melancholy.
— **morning**: agar. aloe. am-c. anac. ant-cr. apis. arg-n. cann-i. lach. mag-m. mag-s. petr. phos. sars. sep. sulph. sul-ac. tarent. tarax.
— — amel. in: carb-an.
— — on rising: hepar.
— — on waking: carb-an. cop. k-ca. nit-ac. petr. phos.
— **afternoon**: alum. calc-s. cast. cocc. con. cop. ign. mang. mur-ac. plat. ruta. sulph. znc.
— — amel.: agar.
— **evening**: agar. am-c. ant-cr. ant-t. bar. bov. calc-s. carb-an. creos. cyc. dig. graph. hipp. ign. k-ca. k-clc. lyc. mag-c. murx. nat-m. nat-p. nit-ac. phos. plat. ran-sc. ruta. senec. sep. stram. sulph. therid.
— — amel.: aloe. cann-s. carb-v. coca. znc.

— **night**: lil-t. tarent.
— **air**, in open: k-ca. mur-ac. sulph.
— — — amel.: plat. rhus. tarent.
— **annoyance**, from least: k-bi.
— **coldness**, with: am-c.
— **eating**, after: anac. ars. chin. *nat-c.* nx-v. ol-an. puls. znc.
— — amel.: clem. tarent.
— **fever** (intermittent) during the chill: ars. calc. *con.* hell. *ign. lyc.* nat-m. phos. plat. selen. sep.
— — during the heat: ars. graph. lyc. *nx-m.* ph-ac. sep.
— — during the sweat: ars. *aur. calc.* con. ign. lyc. nat-m. selen.
— **menses**, before: am-c. calc. caus. con. cyc. ferr. *lyc. nat-m.* nit-ac. *puls.* stann. xanth.
— — during: *am-c.* aur. berb. brom. cact. calc. cimic. cop. ferr. ign. mur-ac. nat-c. *nat-m.* plat. plb. puls. senec. *sep. sil.* thu. znc.
— — — amel.: stann.
— — after: alum. ferr. ustil.
— **music**, from: acon. dig. lyc. *nat-s.*
— **noise**, from: ant-cr. phos.
— **pains**, from the: sars.
— **pollutions**, from: *calad.* con. ham. nat-m. nat-p. ph-ac. sil. sulph.
— **room**, in: plat. rhus. tarent.
— — indoors, becomes lively: ph-ac.
— **society**, in: agar. euph.
— — amel.: bov.
— **solitude**, in: ars. bov. *stram.*
— **twilight**, in: *phos.*
— **walking**, when: acon. therid. thu.
— — in open air: con. *ph-ac.* sep. sulph.

**Memory**, compare Thoughts and Mind.
— **active** (*i. e.*, increased capacity. etc.): acon. agar. alum. anac. ang. arn. ars. aur. bad. *bell.* bov. brom. calc-p. camph. cann-i. cann-s. caps. carb-v. chin. cimic. cob. coca. cocc-c. cocc. coff. croc. cub. cupr. cyc. dig. ether. fluor-ac. glon. grat. hipp. hydr-ac. hyos. lach. lac-ac. laur. lyc. manc. meph. nat-p. nx-m. nx-v. op. oxal-ac. phos. phys. pip-m. plat. plb. puls. raph. rhus. seneg. sil. spig. sulph. tereb. thu. valer. verat. verb. viol-od. znc. ziz.
— — changing from keenness to dullness: rhus.
— — until midnight: *coff.*
— — with lassitude: *aloe.*
— — preventing sleep. See under Sleep.
— **increased** memory: acon. anac. ang. ars. aur. *bell. coff.* croc. cyc. fluor-ac. hyos. lach. lac-ac. op. phos. seneg. spig. valer. viol-od.
— — for things done: cann-i. elæis. sol-t-æ.
— — for things experienced: fluor-ac.
— — for music heard: lyc.
— — for persons: hyos.
— — for things read: bapt. cann-i.
— —for what he does not care to remember: hyos.
— — for things seen: cann-i. seneg.
— **increased** imagination, lively fancy, etc.: acon. agar. anac. bell. cann-i. chin. coca. coff. colch. cyc. dig. glon. lach. meph. op. phos. znc.
— increased inventive faculty, scheming tendency: ang. chin. coff. lach.

**Men**, dread of: aur. bar. plat. puls. sulph.
— See Company.

**Mesmerized**, desires to be: calc. phos.
— seemed as if: œna.

**Mildness**: ambr. anac. ars. asar. aur. bell. bov. caps. carb-an. caus. cic. clem. *cocc.* croc. cupr. euph. euphr. hell. *ign.* iod. k-ca. *lyc.* mang. mosch. mur-ac. nat-c. nat-m. op. ph-ac. plb. *puls.* rhus. sil. stann. stram. sulph. verat. viol-od. znc.

**Mind**, absence of: acon. æsc-h. alum. am-c. am-m. anac. ang. apis. arg. arn. arum-t. asar. aur. bar. bell. bov. calad. calc. cann-s. caps. *caus. cham.* chel. chin. cic. cocc. coff. colch. con. creos. croc. crotal. cyc. dulc. elaps. graph. hell. hepar. hyos. ign. k-bi. k-ca. lac-can. lach. led. *lyc.* mag-c. mang. merc. mezer. mosch. nat-c. nat-m. nit-ac. nx-m. *nx-v.* olnd. op. petr. phos. ph-ac. plat. plb. *puls.* rhod. rhus. ruta. sars. *sep.* sil. spong. stann. sulph. sul-ac. thu. verat. verb. viol-od. viol-tr. znc.
— Compare with Forgetful, Mistakes, etc.
— **periodical** attacks of, short lasting: fluor-ac.
— **diminished** power of, over body: gels. hell.
— — Compare with Awkward.
— **dullness**, of: agn. *alum.* am-c. anac. ant-c. arg-n. asar. ars. bell. calc. cham. chin. con. cyc. graph. *hell.* hyos. *lach. laur. lyc. mezer.* nat-c. *nat-m.* nicc. *nx-v. olnd.* op. *ph-ac.* plb. puls.

*ran-b.* rheum. rhus. sil. spong. *staph.* stram. sulph. thu.
— — See also Weakness.
— **oppression** of: evon. graph. iod. ran-b.
— **weakness** of memory, etc.: acon. agn. ailan. *alum.* ambr. am-c. am-m. *anac.* apis. arg-n. arn. ars. *aur.* bar. *bell.* borax. *bov.* bry. *calc.* camph. cann-s. carb-an. *carb-v.* caus. chel. chin. clem. cocc. coff. colch. con. creos. croc. cupr. cyc. dig. euphr. graph. guai. hell. *hepar. hyos.* ign. ipec. k-ca. *lach. laur. lyc.* mag-c. mang. *merc.* merc-c. mezer. mosch. murx. nat-c. *nat-m.* nit-ac. nx-m. *nx-v.* olnd. *op.* petr. phos. ph-ac. pic-ac. plat. plb. *puls.* rhod. *rhus.* ruta. sabad. sabin. sars. selen. seneg. *sep. sil.* spig. spong. *stann. staph. stram.* stront. *sulph.* sul-ac. thu. valer. *verat.* verb. viol-od. viol-tr. *znc.*
— — See also Mistakes.
— — for **business**: agn. creos. fluor-ac. hyos. k-ca. phos. sabin. selen. sulph. tellur.
— — for **dates**: acon. con. k-bro.
— — **done**, for what has just: acon. agar. aster. absinth. bar. borax. bufo. calad. calc-p. chel. hyos. lac-can. lyc.
— — for what was about to do: agn. bar. bell. calc-p. calc-s. chel. creos. cinnb. fluor-ac. gran. jug-c. sulph.
— — **expressing** one's self, for: bell. cann-s. lac-can. lach. lyc. nx-v. puls. thu.
— — **happened**, for what has: graph. nat-m. rhus. sulph.
— — **heard**, for what has: agar. calc. cann-i. carb-v. hell. hyos. lach. mezer. psor. sulph.
— — **labor**, for mental: acon. aloe. asar. gels. laur. lyc. nat-c. nat-m. ph-ac. pic-ac. selen. sep. sil. sol-m. spig. spong. staph. therid. thu.
— — — — from stupefaction in head: acon. æth. asar. calc. chin. nat-c. nx-m. nx-v. op. plat. rhod. rhus. sep.
— — — — from fatigue: calc. colch. nat-c. nx-v. plat. puls. sep. sil.
— — **letters**, for the names of the: lyc.
— — **names**, for proper: *anac.* croc. fluor-ac. glon. guai. k-bro. lith. *lyc.* merc. olnd. puls. rhus. stram. *sulph.*
— — **persons**, for: acet-ac. agar. ailanth. alcoh. anac. bell. cedr. cham. chlor. croc. hyos. merc. nx-v. op. stram. thu. verat.
— — **read**, for what has: ambr. anac. arn. bell. can-i. coff. colch. chlor. guai. ham. hell. hipp. hyos. lac-can. lach. nat-m. olnd. phos. ph-ac. staph. viol-od.
— — **said**, for what has: arn. bar. cann-i. carb-an. carb-v. colch. croc. hell. hepar. merc. mezer. nx-m. rhod. stram. sulph. verat.
— — for what is about to say: arg-n. arn. atrop. bar. cann-i. carb-an. colch. hell. hydr. lil-t. merc. nx-m. podo. rhod. sulph. verat.
— — **sudden** and periodical: carb-v.
— — **things**, names of, for: *lyc.* rhus.
— — **thought**, for what has just: acon. agar. alum. anac. bell. cann-i. cocc. colch. fluor-ac. hyos. nat-m. ran-sc. stram. verb.
— — **words**, for: agar. arg-n. bar. ham. hydrph. k-bro. lyc.
— — **write**, for what is about to: cann-i. croc. dirc. nat-m. nx-m. rhus.
— **Conditions** of Weakness of Mind.
— — **morning**: anac.
— — **blood**, from rush of: chin. merc. rhus. sulph.
— — **damp** air or dwelling: calc. *carb-v.* puls. *rhus.* sil. verat.
— — **chill**, in a: bar.
— — **emotions**, from: *acon.* op. ph-ac. *staph.*
— — **head**, with stinging in: znc.
— — after injuring head: arn. cic. hyper. merc. rhus.
— — **loss** of fluids, from: *chin.* nx-v. sulph.
— — **mental** exertion, from: aur. calc. lach. nat-c. nat-m. nx-v. ph-ac. pic-ac. puls. sil. sulph.

**Mirth**, Hilarity, Liveliness, etc.: acon. æth. alum. am-c. anac. ang. ant-t. arg. arn. asaf. asar. aur. bar. *bell.* cann-s. caps. carb-an. carb-v. caus. chin. chin-s. cic. cocc. *coff.* con. creos. *croc.* cupr. cyc. ferr. fluor-ac. gamb. graph. hyos. ign. iod. lach. lachn. laur. lyc. mag-c. marum. men. merc. merc-c. mezer. *nat-c.* nat-m. nx-m. *op.* oxal-ac. par. petr. phos. ph-ac. plb. puls. sabad. sars. seneg. sep. spig. spong. stann. staph. *stram.* sulph. sul-ac. tabac. tarax. therid. thu. valer. verb. znc.

— Compare with Cheerful, Laughing, etc.
— **alternating** with Ill-humor. See under Ill-humor.
— — with lachrymose mood: plb. psor. spong. sep.
— — — palpitation: spig.
— **foolish**: acon. bell. calc. carb-v. merc. seneg.
— **Conditions** of Mirth.
— **morning**: con. graph. mag-m. sulph.
— — on waking: chin.
— **afternoon**: ang. cann-s. lyc. merc-i-fl. phos.
— **evening**: am-c. chin. coc-c. ferr. lach. pip-m. sulph. viol-tr.
— — ill-humor during the day, merry in the evening: sulph. viol-tr.
— — noon, low-spirited at, lively in evening or vice versa: znc.
— **night**: chin. naja.
— **emission**, after an: pip-m.
— **weeping**, after: *plat.*
**Misanthropy**: *am-m.* anac. *aur.* bar. *calc.* cic. hyos. *led.* lyc. nat-c. *phos.* plat. puls. *stann.*
**Mischievous**: aloe. bar. cupr. lach. nx-v.
**Miserly**: ars. bry. calc. cina. coloc. *lyc.* nat-c. *puls.* rheum. *sep.*
**Mistakes**, makes in calculating: ailan. am-c. merc. nx-v. sumb.
— **localities**, in: bry. *glon.* hura. lach. nx-m. par. petr. sulph. valer. verat.
— **measure** and weight and gives wrong answers: nx-v.
— **names**: dios. *stram.*
— **reading**: cham. hyos. lyc. merc. sil. stann.
— **speaking**: acet-ac. agar. alum. am-c. arg-n. bov. calc. cann-s. canth. caus. *chin.* chin-s. coca. con. crotal. cupr. cyc. dios. graph. hæmat. ham. hepar. hyos. ign. k-bro. k-ca. lac-can. lach. lil-t. lyc. mang. merc. murx. *nat-m.* nx-m. nx-v. osm. ph-ac. rhus-r. secale. selen. sep. sil. stram. sulph. sul-ac. thu.
— — worse after exertion: agar.
— — utters wrong syllables: *lyc.* selen.
— — gives wrong answers: phos.
— **spelling**, in: fluor-ac. lach. lac-ac. lac-can. lyc. nx-m. nx-v. stram. sulph.
— **time**, in: anac. cic. cocc. croc. fluor-ac. hura. *lach.* nx-v. petr. therid.
— — See also Time.
— **words**, misplacing: alum. am-c. arn. bov. *calc.* cann-s. caus. cham. chin. cocc. con. crotal. cyc. graph. hepar. hyos. k-ca. lac-can. lach. lyc. merc. nat-c. nat-m. *nx-v.* osm. puls. rhod. sep. sil. stram. sulph. thu.
— — omits: benz-ac. *cham.* nx-v. rhod.
— — reverses: *chin.*
— in **work**: acet-ac. bell. chin-s. nat-c. nx-v. ruta. selen.
— in **writing**: am-c. benz-ac. bov. calc-p. cann-i. cann-s. carb-an. cham. chin. chin-s. chro-ac. croc. crotal. dios. fluor-ac. graph. hepar. hydr. ign. k-bro. lac-can. *lach.* lac-ac. *lyc.* nat-c. nat-m. nx-m. nx-v. ptel. puls. rhod. rhus. sep. sil. *thu.*
**Moaning**: acon. *apis.* *ars.* bell. cann-i. canth. caus. *cham.* *cic.* cina. cocc. coff. colch. creos. eup-per. gels. graph. hell. hyos. ign. ipec. lach. merc. mur-ac. naja. nx-v. op. oxal-ac. *phos.* phyt. rheum. sars. squil. stram. sulph. sul-ac. tabac. tanac. tarent. verat. znc.
— Compare with Groaning.
— at **night**: ars. cupr. hepar. tarent.
— **sleep**, during: aloe. ars. bry. calad. cham. lach. mur-ac. nx-v. op. podo. stann. sulph.
— **chill**, during: eup-per.
— **constant** moaning and gasping for air: phyt.
— **fever**, during: acon. cham. eup-per. lach. puls.
— **hemicrania**, with: cop.
— **ill-humor**, with: *cham.*
— **pain**, during: bad. eup-per. hura.
**Mocking**: ars. chin. ipec. lach. plat. par.
— his relations: secale.
**Monomania**: acon. carb-v. *ign.* nx-m. puls. *sil.* stram. thu.
**Moping**. See Sadness.
**Moral** feeling, want of: *anac.* bism. con. hyos. *laur.* op. sabad.
**Morose**. See Melancholy, Sadness, etc.
**Murder**, desire to: ars. camph. chin. *hepar.* *hyos.* lach. plat. stram. thea.
— — — her husband, when alone with him: nx-v.
— dread of being murdered: op. phos. stram.
— — — — — when dreaming: am-m. guai. ign. k-iod. lact. lyc. merc. znc.
— and homicide, continually before the mind: calc. op. phos. stram.

— continual thoughts of, when dreaming: am-m. calc. carb-an. guai. ign. k-iod. lact. led. lyc. merc. nat-c. *nat-m.* ol-an. *petr.* rhus. sil. spong. *staph.* znc.
— See also Killed.
**Music**, averse to: *sabin.*
— See also under Sensitiveness.
**Muttering**: apis. arn. *bell.* calad. colch. dulc. ether. hyos. lach. lyc. nx-v. ph-ac. plb. sil. *stram.* sul-ac. tabac. tarax. vip.
— See under Delirium.
— to himself: hyos. tabac.
— in sleep: ars. camph. indg. merc.
— unintelligible: *ars.* cann-s. hyos. *stram.*
**Naked**, wants to be: bell. *hyos.* phos. phyt.
— — — in delirium: merc.
— — — Compare Covering.
**Nervous.** See Anxiety, Fretful, Restlessness, etc.
**Nobody**, feels as if he were: ang.
**Noise**, averse to. See under Sensitive.
**Obscene**, in speech: lil-t.
— See Lewdness.
**Obstacles**, wants them to be removed, in sleep: cham.
**Obstinate**: *acon. alum.* am-c. anac. arn. ars. bell. bry. *calc.* canth. *caps.* carb-an. carb-v. caus. cham. chel. chin. creos. dig. dros. ferr. guai. hepar. *ign.* k-ca. k-iod. lach. lyc. merc. *nit-ac. nx-v.* phos. ph-ac. secale. sil. spong. stram. *sulph.* thu. viol-od. znc.
— children, yet cry when kindly spoken to: sil.
— on appearance of menses: *cham.*
**Occupied**, or busy, general amelioration when: agar. bar. chin. croc. ferr. *helon.* NAT-C. pip-m. SEP. stram. thu.
**Offended**, easily: acon. alum. ars. aur. bov. calc. camph. cann-s. caps. carb-v. caus. cham. chin. cina. cinnb. cocc. cyc. dros. lyc. nx-v. phos. plat. puls. ran-b. sars. sep. spig. sulph. verat.
— Compare with Sensitive.
**Oppression**, mental: cann-i. psor. ran-b. ran-sc. tilia.
— Compare with Melancholy, etc.
**Over-sensitive.** See Sensitive and compare with Intolerance.
**Passionate**: ars. aur. cann-i. canth. coff. hyos. k-iod. laur. lyc. olnd. petr. phos. nat-m. nat-s. nx-v. sep. stram. *sulph.* tarent.
— at every trifle: nat-m. nat-s. phos. ph-ac. sumb.
**Patient**: mag-m. phos.
**Peevish.** See Fretful, Ill-humor, etc.
**Pertinacity**: caps. dros. stram.
**Perverse**: aur. cham. croc. hura. k-ca. nitr. thu.
— See also Obstinate.
**Petulant.** See Fretful.
**Phlegmatic.** See Indifference.
**Picking.** See under Gestures.
**Pities** herself: agar.
**Plans**, making many: anac. ang. *chin.* coff. olnd.
— gigantic: op.
— revengeful: agar.
**Playful**: aloe. cimic. cocc. elaps. lach. men. naja. oxal-ac. seneg. tarent.
— alternating with melancholy: psor.
— plays with the buttons of his clothes: mosch.
— indisposition to play, in children *bar.*
— desires to play in the grass: elaps.
**Pleasure**: ang. cann-i. tilia.
— during wakefulness: secale.
— on waking from a dream of murder: thea.
— in voluptuous ideas only: bell.
— in nothing: hura. ipec. mezer. staph. *sulph.* therid.
— — — See Discontented.
— in his own talking: par. stram.
**Poor**, thinks is: bell. hepar. nx-v. *sep.* valer.
**Positiveness**: camph. caus. ferr. lach. merc.
— Compare with Obstinate.
**Possessed**, a condition as if: anac. hyos.
— Compare with Delusions.
**Praying**: *aur. bell.* op. puls. stram. *verat.*
**Precision** of mind increased: ferr-p.
**Precocity**: merc.
**Pre-occupied.** See Mind, absence of.
**Pride**: alum. arn. chin. cic. cupr. ferr. hyos. ipec. *lach.* lyc. par. *plat.* stram. sulph. verat.
**Prophesying**: *acon.* agar.
— **predicts** the time of death: ACON.
**Prostration.** See Weakness.
**Pulls**, desires to pull one's hair: bell. tarent.
— one's nose in the street: merc.
— one's teeth: bell.
**Puns**, disposed to make: cann-i.
**Quarrelsome**: acon. agar. alum. ambr.

am-c. anac. ant-t. arn. ars. *aur.* bar. bell. bov. *brom.* *bry.* calc. *camph.* canth. caps. *caus.* *cham.* chin. croc. crotal. dig. dulc. elaps. ferr. *hyos.* *ign.* k-iod. lach. lyc. merc. merl. mezer. mosch. nat-c. nat-m. nat-s. nicc. nit-ac. *nx-v.* olnd. petr. plat. psor. *ran-b.* ratan. ruta. seneg. sep. spong. staph. stram. stront. sulph. thea. thu. tilia. verat. *verat-v.* viol-tr. znc.
— **alternating** with gayety and laughter: croc. spong.
— — — care and discontent: ran-b.
— — — singing: croc.
— desires to quarrel: atrop. hyos. sul-[ac.
— **morning**: petr. psor. ran-b.
— **afternoon**: dulc.
— **evening**: am-c. nat-m. nicc. psor. sil.
— in **sleep**: ars. rheum.

**Quiet** disposition: asar. caps. cic. clem. cocc. euph. lyc. mang. mur-ac. nx-v. plat. puls. sars. sil. stann. viol-tr. znc.
— cannot be quieted: CINA.
— only by being carried: CHAM.
— wants to be: *bry.* cann-i. coca. cupr-s. dios. euph. *gels.*
— — — during chill: ars.
— — — desires repose and tranquillity: nx-v.

**Quick** to act: coff.

**Rage**, fury: acon. æth. agar. ant-t. arg-n. ars. bar. *bell.* camph. cann-i. cann-s. *canth.* cham cocc. colch. croc. cupr. dros. *hyos.* k-ca. lach. lyc. merc. nat-m. nit-ac. op. phos. plb. puls. ruta. sabad. secale. seneg. *stram.* verat.
— See Anger, etc.
— **alternating** with convulsions: stram.
— **convulsive**: bell. stram.
— after insults: sang. stram.
— bright objects aggr.: canth.
— during menses: acon.
— when trying to drink, or from touching larynx: canth.
— with staring looks: bell.
— cold application to head amel.: sabad.
— epilepsy, after: arg.
— pulls hair of bystanders: *bell.*
— does not know his relations: bell.
— about trifles: cann-s.
— violent: bell. hyos.

**Rashness**: caps. men. puls.

**Reading**, averse to: acon. brom. clem. coca. corn. cyc. hydr. k-bi. lil-t. phys.
— desires to be read to: chin. clem.
— makes mistakes in: cham. hyos. lyc. merc. sil. stann.
— aversion to, amel. in open air: oxal-ac.

**Reason**. See Mind, Thought, etc.
— loss of, and see Insanity, Mania, etc.

**Recognize**, does not, his relations: bell. hyos. merc. verat.

**Recollect**. See Mind, Memory, etc.

**Reflection**. See Thoughts.

**Refuses** or rejects things which were desired: ars. bell. bry. *cham.* chin. cina. dulc. hepar. puls. rheum. staph.

**Religious** afflictions: alum. am-c. *ars.* *aur.* *bell.* carb-v. caus. cham. cina. coff. con. croc. cyc. dig. ferr. graph. HYOS. *ign.* LACH. lil-t. *lyc.* merc. nx-v. plat. *puls.* ruta. sabad. selen. sil. *stram.* *sulph.* *verat.* znc.
— **feeling**, want of: anac. coloc.
— See also under Anxiety, Despair, Doubtful, etc.

**Remorse**: alum. am-c. *ars.* aur. *bell.* carb-v. caus. cham. cina. *cocc.* coff. con. *cupr.* cyc. dig. ferr. graph. hyos. *ign.* lach. merc. nx-v. puls. ruta. sabad. selen. sil. stram. sulph. *verat.* *znc.*
— repents quickly: croc. olnd.

**Reproaches**: acon. *ars.* caps. chin. cic. hyos. ign. *lach.* lyc. mezer. nat-m. *nx-v.* par. ph-ac. sep. verat.
— himself: calc-p. hura. hyos.

**Repulsive** mood: acon. alum. ambr. ant-cr. arn. ars. aur. bell. camph. caps. caus. con. croc. hepar. ign. ipec. k-ca. lact. laur. led. lyc. mag-c. mag-m. *merc.* nit-ac. nx-v. petr. phos. plb. *puls.* samb. sars. sil. spong. sulph. thu.

**Resentment**: berb. mang. nit-ac.

**Reserved**: alum. arg-n. ars. aur. bell. bism. calc. caps. carb-an. caus. cham. chin. clem. coloc. cyc. dros. euph. euphr. grat. *hell.* *hyos.* *ign.* ipec. lach. lyc. mag-c. *mang.* *mur-ac.* nat-m. nit-ac. nx-v. ol-an. op. petr. phos. plat. plb. *puls.* rheum. sabad. sabin. spong. *stann.* verat.

**Resistance**: caps. nx-v.
— to everything done for him: bell.
— a feeling of to be overcome on moving: *secale.*

— See Obstinate, etc.
**Resolve**, difficult to: mezer.
— impossible: k-bro. tabac.
— slow of: graph.
**Resolute**. See Boldness.
**Rest**, cannot when things are not in proper place: anac. *ars.*
**Restlessness**, restless, etc.: abies-c. abies-n. abrot. ACON. acon-c. æth. agar. agar-ph. ailan. alco. all-c. aloe. alum. almn. ambr. am-c. ammc. aml-n. *anac.* anthr. ant-cr. ant-s. *ant-t. apis.* apoc. apom. arg. arg-n. arn. ARS. ars-i. ars-h. *asaf.* asar. asc-t. aster. atro. *aur.* bad. BAPT. bar. BELL. bism. borax. bov. bry. cadm. cai. calad. calc. calc-p. calc-s. calen. calo. CAMPH. cann-i. cann-s. canth. carb-an. carb-v. carbn-o. carbn-s. casc. castor. caul. caus. cedr. ceph. cer-b. cerv. *cham.* chel. chim. chin. chin-s. chlf. chlo. chr-ac. cimic. cina. cinnb. cist. CIT-V. clem. cob. coca. *cocc.* cocc-c. cod. coff. coff-t. coffn. colch. *coloc.* coll. com. con. *cop.* coral. corn. creos. croc. crot-c. crotal. croton. cub. *cupr.* cupr-ac. cupr-ar. cupr-s. cur. cyc. daph. *dig.* dios. dirc. dor. dros. dub. dulc. elaps. erig. eugen. euphr. eupi. ery-a. eth. ferr. ferr-i. ferr-m. fluor-ac. gal-ac. gels. gent. gins. *graph.* guai. guara. hæmat. ham. hell. helon. hipp. hydr-ac. hydrph. HYOS. hyper. ign. indg. *iod.* ipec. iris. jabor. jatr. k-bi. k-bro. k-ca. k-clc. k-cy. k-iod. k-sul. kalm. lab. *lac-can. lach.* lachn. lac-ac. lact. lam. laur. *led.* lepi. lil-t. lobel. lyc. mac. mag-c. mag-m. mag-s. manc. mand. mang. meph. MERC. *merc-c.* merc-d. merc-i-r. merc-m. merc-s. merl. mezer. mill. morph. mosch. mur-ac. myric. naja. nat-ar. *nat-c.* nat-m. nat-s. nicc. nicot. *nit-ac.* nitr. nit-m-ac. nit-ox. nuph. nx-m. *nx-v.* œna. olnd. op. osm. oxal-ac. petr. phos. ph-ac. phys. phyt. plan. plat. PLB. prun. psor. ptel. PULS. puls-n. ran-b. rheum. rhod. RHUS. rhus-v. rumx. ruta. sabad. sabin. samb. sant. sarr. scor. scut. secale. *sep. sil.* sol-m. sol-n. sol-t. spig. *stann. staph.* STRAM. SULPH. sul-ac. sumb. *tabac.* tarax. *tarent.* tax. *tell. thu.* ton. trom. upa. vac. *valer.* verat. vinc. viol-od. vip. wies. yuc. zing. znc-a. znc-m. znc-s.
— **alternating** with sleepiness and stupor, during fever: *ars.*
— **anxious**; with anxiety, etc.: *acon.* alum. ambr. anac. arg. ARS. asaf. aspar. *aur.* bell. bov. bry. calc. calc-p. camph. canth. caps. carb-an. carb-v. caus. *cham.* chin. chin-s. cimic. clem. coff. croc. crotal. dros. graph. hell. hepar. lact. lam. men. merc. nat-c. nat-m. nit-ac. nx-v. op. phos. ph-ac. plat. puls. rhus. ruta. sabad. sep. sil. spig. spong. staph. *tabac.* valer. verat. wies. znc.
— — compelling rapid walking: *arg-n.* ars. lil-t. sul-ac.
— **fatigue**, with: ARS.
— **hysterical**: asaf. aur.
— **internal**: acon. agar. atro. ars. carb-an. carl. chel. eupi. gins. lobel. lyc. mag-c. mag-m. mag-s. meph. nat-m. op. par. plb. phos. ph-ac. ran-b. rheum. RHUS. sep. SIL.
— — as if would beat about herself with hands and feet: lyc.
— — morning when waking: sep.
— — evening in bed: eupi.
— — night on waking, with headache: par. [*ars.*
— — head, with stupefied feeling in:
— — toothache, with: mang.
— **mental** (so stated): *acon.* agar. ang. ant-t. apis. arn. arum-i. calc. caps. cham. chel. dulc. hydrph. iod. k-ca. merc. plan. *plat.* ptel. *puls.* ratan. RHUS. thu. verat. vip. znc.
— — Compare with Anxiety.
— — during uterine hemorrhage.
— **nervous**: *acon.* apis. *cimic.* cupr. nat-ar. phyt. plb. *sep. sulph.* tabac.
— — during chill: acon.
— **pacing** back and forwards: plan.
— **periodical**: *ars.*
— — every third day: anac.
— **rage**, ending in a: canth.
— **tremulous**: arn. *plat.*
— **wandering**, night, from pain: ferr.
— **whining**: cham.
**Conditions** of Restlessness.
— **day**, during: ambr. nat-c. nat-m. pip-m. plan. rhus. staph.
— **morning**: ailan. fago. gamb. ir-fœ. k-bro. *lyc.* nat-m. myric. phys. sulph. thu. upa.
— — aggr. in: *gels.* iod.
— — in bed: ph-ac.
— — on waking: dulc. hyper.
— **forenoon**: calad. cimic. fago. hydrph. thu.
— **noon**: bell. hydrph.

— **afternoon**: anac. apis. aur. calc-s. carb-v. caul. cimic. coloc. dios. fago. jug-c. merc-s. naja. nicc. staph. tabac. thu. upa.
— **evening**: acon. agar. *alum. am-c.* ars. bov. calc. calc-s. carb-v. caul. *caus.* chin-s. clem. dios. equ. eth. fago. guare. hepar. jabor. laur. lyc. mag-c. mag-m. meph. *merc.* mur-ac. nat-c. nicc. nx-v. phos. ph-ac. phys. *rumx.* ruta. scut. sulph. thu. zing.
— — aggr. in: carb-v. laur. merc. nx-v. phos.
— — in bed: lyc. mag-m. nx-v. phos. sabin. sep. thu.
— — at 8 P. M.: MERC.
— — 4 to 6 P. M.: carb-v.
— **midnight**, at: nat-m.
— — on waking: plat.
— — before: alum. cot. sars. senec.
— — after: dios. lyc. merc-i-r. rhus-v. sil. sulph. znc.
— **night**: abies-c. abies-n. abrot. acon. acon-c. am-cau. am-m. anac. ang. anil. ant-n. anthr. ant-ox. ant-t. apis. apoc. *arg.* arg-n. ars. *ars-i.* asaf. asc-t. aster. atro. aur. aur-s. bad. *bapt. bell.* bism. bov. bry. cact. cai. calc. calc-cau. calen. calo. camp. canth. carb-ac. carb-an. carb-c. carbn. carbn-s. carb-v. card-m. cast-v. castor. *caul.* CAUS. cedr. *cham.* chr-ac. cimic. cina. cinnb. cist. *cit-v.* clem. coca. cocc-c. coloc. com. coral. corn. creos. croton. cupr-s. *cyc.* dig. dios. dirc. equ. erig. eupi. euphr. fago. ferr-i. fluor-ac. form. fran. gal-ac. gels. gett. glon. gnap. graph. hall. hura. hydrs. hydrph. hyper. iber. *ign.* indg. indm. *iris.* jac. jatr. jug-c. k-ar. k-bi. lach. lyc. mag-c. mag-m. mang. *marum. meni.* ment-pu. merc. *merc-c.* merc-cy. merc-m. merc-s. morph. mosch. mur-ac. myric. nat-c. nat-m. nat-s. nicc. nicot. nit-ac. nx-m. nx-v. nym. op. osm. ost. oxal-ac. ped. petr. *phos.* ph-ac. phys. phyt. pip-m. plan. *podo.* ptel. puls-n. *ran-b.* RHUS. rhus-v. rumx. ruta. sang. sap. senec. *sep.* sil. sol-t-æ. spong. spira. stram. stry. sulph. sul-i. sul-ac. tabac. tarax. thea. thu. ustil. *valer.* verat. verat-v. verb. vesp. vip. yuc. znc.
— — Compare with Sleep, restless.
— — 1 A. M.: gett. nat-ar. phos.
— — 2 A. M.: ambr. com. ferr. graph. mag-m. myric. zing.
— — 3 A. M.: agar. cimic. cocc-c. creos. nat-ar. nat-m.
— — — at 3 A. M., everything feels sore, must move about: nicc.
— — 4 A. M.: clem. creos. nit-ac. trom. wild.
— — 5 A. M.: tarent.
— — aggr. at: *ars.* plb. rhus.
— **abdomen**, from constriction in: mosch.
— — distention in, at night: calc-s. caus. chel. lyc. mag-c. valer.
— — gastralgia, at night: tarent.
— — hypochondrium, from pain in: calc-fl.
— — from pain in: cob.
— — stomach, from pain in: *canth.* cob.
— **air**, in open, amel.: laur. lyc.
— — after walking in, amel.: graph.
— **alone**, when: all-s. mezer. *phos.*
— **anxiety**, from. See Anxious Restlessness.
— **back**, during tired aching in: calc-fl.
— **bed**, driving out of: ARS. bry. *carb-v.* caus. *cham.* chin. chin-s. con. *graph. hepar.* hyos. *lyc. mag-c.* nat-m. nx-v. puls. *rhus.* sep. sil. therid.
— — heat of, from: op.
— — wants to go from one bed to another: *ars.* bell. *calc.* cham. cina. *hyos.* mezer. rhus. sep. verat.
— — tossing about in: *acon.* alum. apis. *ars.* asaf. bapt. bell. bry. calc. *castor. cham.* cic. *cina.* cist. clem. cocc. coral. creos. croton. ferr. ferr-m, guai. hell. ign. lach. led. lyc. merc. mur-ac. nat-c. nat-m. op. par. phos. puls. *ran-sc.* rheum. rhus. senn. sep. squil. staph. sulph. valer. verat.
— **chest**, from congestion in: sep.
— — from heat rising up into mouth from chest: *nx-v.*
— **children**, in: ant-t. borax. *cham. jalap.* rheum.
— — relieved by being carried about: ant-t. ars. *cham.* cina. k-ca.
— **coffee**, after: bart.
— **coition**, after, at night: cop.
— **delirium**, during: *ars.*
— **dinner**, after: am-m. nat-c. ruta. thu.
— — amel. after: thu.
— **dreams**, from: ph-ac.
— **drink**, at the sight of: bell.
— **diarrhœa**, during: hydrs.

— **eating**, when: borax. petr.
— — after: am-m. bar. chin. cinnb. lach. nx-m. nx-v. petr. phos. ph-ac. rhod. sulph.
— **eye**, on closing at night aggr.: sep.
— **fainting**, followed by: calc.
— **fever**, during the heat: *acon.* am-c. ant-t. *arn.* ARS. atro. bapt. *bar.* bell. caps. *cham.* china. cina. con. cub. *gels.* hyper. ipec. lachn. mag-c. mag-m. mosch. mur-ac. *op.* plant. PULS. rheum. RHUS. sabin. *secale.* spong. staph. *sulph.* thu. valer.
— — after heat at night: ph-ac. puls. sep.
— — **chill**, during the: anac. ARS. asaf. bell. borax. cann-s. caps. carb-v. creos. eup-pur. mezer. plant. plat. rhus. spig.
— — — at beginning of chill: *lach.* phos.
— — **sweat**, during: bry. graph. lachn. samb.
— **fit**, after a: œna.
— **headache**, during: anac. arg-n. *ars.* bry. cadm. calad. canth. cham. chin. daph. gent. ign. k-iod. *lach. lyc.* morp. naja. nx-m. ran-b. ruta. sil. vip-t.
— — from pain in forehead, at night: cyc.
— **heart**, during palpitation: æth. aspar.
— **hips**, from bruised pain in, at night: form.
— **knee**, from digging in: spig.
— — from itching in: mang.
— **lochia**, with: coloc. rhus.
— **lying** down, when: aur. cit-v. mag-m. nx-v.
— — on back, aggr.; on side amel.: calc-p.
— **menses**, before: acon. con. creos. k-ca. lyc. mang. *nx-v.* sulph.
— — during: *acon.* am-c. ant-t. apis. *ars.* bell. borax. calc. *cham.* cocc. *coff.* croc. gels. hyos. ign. ipec. k-ca. mag-m merc. nat-c. nit-ac. *nx-v.* op. phos. plat. puls. *rhus.* secale. *sep.* sulph. thu.
— — during suppressed menses: ars. cimic k-ca. nicc. nx-v. rhus. sep. znc.
— — during metrorrhagia: *acon. apis.* cham. hyos. stram.
— — after: mag-c.
— **mental** labor, during: borax. nat-c.
— **music**, from: tarent. [cai.
— **micturition**, during desire for:
— **nausea**, during: borax. cina. phos.
— **pain**, from: *ars. cham. coff.* coloc. dios. ferr. lyc. *mag-c.* mang. plb. *puls. rhus.* tabac.
— **paroxysms**, during: plb.
— **parts** affected, in: arn. chin. ferr.
— **pregnancy**, during: acon. verat.
— **position**, constantly changing: *ars.* bell. canth. cham. cupr. iod. *puls. rhus.*
— — every position becomes annoying: bism.
— — lying long in one position, aggr.: nat-s. *puls.*
— **reading**, while: sumb.
— **respiration**, with difficult: prun.
— **rest**, when at: creos. plat. rhus. puls.
— **rising**, on: atro. fago. ptel.
— — from a seat: caus.
— **room**, in: lyc.
— **sleep**, before: thu.
— — during. See under Sleep, restless.
— **sleeplessness**, with: puls.
— — See also under Sleep, prevented by restlessness.
— **sitting**, while: alum. cact. *caus.* lyc. mag-c. nat-m. plan. *sil.* sulph.
— **smoking**, when: calad.
— **stool**, during: bell.
— **storm**, during a: gels. nat-c. nat-m. phos. psor.
— **stretching** backward, amel.: borax.
— **sweat**, amel.: *sulph.*
— **talking**, after: ambr. borax.
— **thoughts**, from lascivious: graph.
— **touch**, on: œna.
— **toothache**, during: sep.
— — from boring, tearing in: sulph.
— **tosses** about greatly, raises up in delirium: cham. cina. hell. stram.
— **vomiting**, colic and suppressed menses, with: nicc.
— **waking**, on: chin. cina. dulc. hyper. sep. *sil.* stann.
— **walking**, when: ambr. caus. pæon. ran-b. thu.
— — amel.: dios. nat-m. nicc.
— — in open air amel.: graph.
— **working**, when: cit-v. graph. vos.

**Retiring** disposition: æth. *indg.* ipec. olnd.
— Compare Reserved.

**Revengeful**: agar. am-c. calc. hydr. lach. mang. nat-m. nit-ac. op.
— Compare with Resentment.

**Reverence** for those around: ham.
**Ridicule**, mania to: acon. hyos. lach. nx-v. verat.
**Riding** in carriage, averse to: psor.
**Risus** sardonicus. See under Laughter.
**Rouses**, with difficulty: bell. lyc. op. selen. sul-ac.
**Roving** about naked: hyos.
— — wrapped in fur, in summer: hyos.
— — senseless, insane: bell. canth. coff. hyos. *nx-v.* sabad. stram. verat.
**Rudeness**: ambr. eugen. hell. *hyos.* lyc. nx-m. nx-v. op. par. phos. stram. verat.
**Sadness**, dejection, etc.: abrot. acet-ac. acon. æsc-h. agar. *agn.* ailan. *ambr.* aloe. *alum.* am-c. am-m. anac. apis. apoc. arg-n. *ars.* arum-t. asaf. asar. AUR. aur-m. bapt. *bar.* bell. berb. bov. brom. bry. *cact. calc.* calc-p. camph. cann-i. *cann-s.* canth. CARB-AN. *carb-v. caus.* cham. chel. *chin. cic.* CIMIC. cina. cinnb. *clem.* coca. *cocc.* coff. colch. coloc. con. creos. *croc.* crotal. croton. cupr. *cyc.* dig. dros. dulc. eugen. eup-pur. euph. euphr. ferr. fluor-ac. gels. graph. grat. guai. hæmat. ham. hell. helon. hepar. hura. hydr. hyos. hyper. *ign. indg. iod.* ipec. k-bi. k-bro. k-ca. k-clc. k-iod. lac-can. lach. lachn. lact. lam. laur. lept. lil-t. *lyc.* mag-c. mag-m. mag-s. men. merc. merc-i-r. merl. *mezer.* mosch. murx. mur-ac. naja. NAT-C. *nat-m.* nat-s. nitr. *nit-ac.* nx-v. olnd. *ol-an. petr.* phel. phos. *ph-ac.* phyt. pic-ac. *plat.* plb. prun. PSOR. ptel. PULS. ran-sc. raph. rheum. rhod. RHUS. *rhus-v.* ruta. sabin. sars. secale. senec. seneg. *sep. sil.* spig. spong. stann. *staph.* stram. stront. SULPH. sul-ac. tabac. thea. thu. tilia. tong. tril. ustil. valer. verat. viol-tr. znc. ziz.
— Compare with Anxiety, Melancholy, etc.
— **alternating** with cheerfulness: acon. agar. asar. canth. carb-an. *chin.* clem. croc. ferr. fluor-ac. gels. hell. ign. iod. lyc. nat-c. nit-ac. nx-m. plat. psor. senec. sep. spig. tarent. znc. ziz.
— **anxious**: cop.
— **causeless**: phos. tarent.
— **criminal**, as if he were: cyc. sabad.
— **future**, about the: anac. arn. bar. caus. chel. cic. con. dig. dros. dulc. k-ca. lach. mang. nat-c. nat-m. phos. ph-ac. rhus. spig. stann. sulph. thu.
— — See also under Anxiety.
— **health**, about: *sep.*
— — great tiredness and laziness, with a feeling of deep-seated inward trouble, which makes him sad: sabin.
— **insult**, as if from: *cocc.*
— **misfortune**, as if from: chin-s. phel. phos.
— **periodical**: *aur.* cop.
— **quiet**: ign. nx-v.
— **suicidal**: op. psor.
**Conditions** of Sadness.
— **morning**: alum. am-c. cann-i. carb-an. caus. dulc. graph. hyper. lach. nit-ac. ol-an. petr. phel. phos. *plat.* puls. rhus. sul-ac. znc.
— — on waking: ars. carb-an. ign. *lach.* phos. ph-ac. tarent.
— — after waking: anac. ant-cr. cop. hipp. nx-m. phel. ptel. thu.
— **noon**, at, lively; in evening sad, or vice versa: *znc.*
— **afternoon**: æth. ant-t. calc-s. carb-an. cast. graph. grat. hydr-ac. mang. myric. phos. thu.
— **evening**: am-c. ant-cr. ars. bar. bov. calc. carb-an. cast. creos. dig. ferr. graph. hepar. ign. k-bi. k-ca. lact. lyc. mag-c. murx. naja. nat-p. nit-ac. phos. *plat.* puls. ran-sc. ruta. senec. seneg. *sep.* stram. sulph. verat. znc.
— **night**: dulc.
— — and day: caus.
— — in bed: ars. graph. stram. sulph.
— — in twilight: *phos.*
— — amel. at: am-c. tarent.
— **air**, in open: æth. k-ca. sabin.
— **alone**, when: æth. aur. bov.
— **anger**, after: plat.
— **annoyance**, after: k-bi.
— **business**, when thinking of, morning: *puls.*
— **chill**, during. See Fever, chilly stage of.
— **colic**, with: plb.
— **complaining**, amel.: tabac.
— **consolation** aggr.: nat-m.
— **diarrhœa**, during: merc.
— **disappiontment**, after: dig.
— **domestic** affairs, after: viol-tr.
— **eating**, after: anac. ars. asaf. canth. hyos. nx-v. *puls.* znc.
— — amel. evening: tarent.
— **emissions**, from: ham. nat-p.

— **exertion**, amel.: ferr.
— **fever**, from: hipp. tarent.
— — (intermittent) during the chill: ACON. *apis*. calc. cann-s. cham. CHIN. cocc. CON. *cyc*. *graph*. IGN. lach. lyc. merc. NAT-M. nit-ac. nx-v. plat. *puls*. rhus. sep. spig. staph.
— — during the heat: ACON. apis. *ars*. *bell*. bry. chin. cocc. *con*. *dig*. graph. ign. lyc. *nat-c*. NAT-M. nx-m. *phos*. ph-ac. plat. puls. *rhus*. sep. *sil*. staph. sulph.
— — during the sweat: *acon*. *apis*. bell. bry. *calc*. *chin*. CON. graph. ign. *nat*-m. nit-ac. nx-v. puls. *rhus*. *sep*. *sulph*.
— **headache**, during: crotal. naja.
— **house**, in: *plat*. *rhus*. tarent.
— **menses**, before: *caus*. lyc. manc. nat-m. stann.
— — during: am-c. cop. ferr. mag-m. mur-ac. nat-m. nit-ac. sep.
— — — first menses: hell.
— — after: ferr.
— — amel: *cyc*. mac.
— **music**, from: acon. dig. lyc. nat-s. tarent.
— **nausea**, with: *sang*.
— **pain**, from: sars.
— **palpitation**, with: *nat-m*. nx-m.
— **pregnancy**, in: lach.
— **pressure** about chest, from: graph.
— **respiration**, with impeded: ant-cr. lach. laur. sep. tabac.
— **sexual** excitement, after: tarent.
— **stories**, from sad: cic.
— **sunshine**, in: stram.
— — amel.: plat.
— **supper**, after, aggr.: nx-v.
— — amel.: am-c.
— **trifles**, about: agar. bar-ac. graph.
— **vertigo**, during: hydr. phos.
— **waking**, on: lach. plat. raph. tarent.
— — See also under Morning.
— **walking**, when: con. tabac.
— — amel.: cop.
— — in open air: ant-cr. coff. con. *ph-ac*. sep. sulph. tabac.
— — — — only when, the longer he walks the worse he gets: *ph-ac*.
— — — — aggr.: sep.
— — — — amel.: rhus.
— **weather**, in cloudy: am-c.
— **weeping**, amel.: dig. phos.
**Scolding**. See Abusive.
**Scorn**. See Contempt.
**Scratches** with hands: stram.
**Scream**. See Crying out, Shrieking, also Weeping.
**Scrupulous**: ang. apis. aur. *bar*. bry. cham. chin. cyc. hyos. ign. mezer. mur-ac. nat-c. *nx-v*. sep. sil. spig. stram. sulph. thu. *verat*.
**Searching** on floor: plb.
— at night, for thieves: ars.
— — — — — after having dreamt of them: nat-m.
**Secrets**, divulges: agar. alcoh.
**Self-confidence**, want of: *anac*. ang. aur. *bar*. *bry*. canth. *chin*. ign. iod. *k-ca*. lyc. mur-ac. olnd. *puls*. rhus. *sil*. stram. tabac. therid.
— self-accusation: cyc.
— self-contradiction: anac.
— self-control, want of: lach.
— — possession: mosch. nat-c.
— — willed. See Obstinate.
**Senses**, acute: ars. bar. *bell*. cann-i. caps. clem. *coff*. hydr-ac. nx-v. *op*. thea.
— **confused**: arg-n. bell. lil-t. mang.
— **dullness**, of: acon. agar. agn. alum. ambr. am-c. anac. ant-t. *arn*. ars. asar. aur. *bell*. bov. bry. calc. camph. canth. caps. caus. chel. cic. con. cyc. dig. dulc. hell. *hyos*. k-ca. ign. iod. lach. lact. laur. led. lyc. mag-c. men. merc. mezer. mosch. *nat-m*. nit-ac. nx-m. nx-v. olnd. ol-an. *op*. petr. phos. ph-ac. plb. ran-sc. rhod. rhus. sabad. *secale*. selen. sil. stann. staph. stram. *sulph*. tabac. therid. verat. znc.
— **vanishing** of: anac. ant-t. asar. ars. bell. borax. bov. bry. calc. camph. canth. carb-an. cham. chel. cic. coff. creos. cupr. glon. graph. hepar. hyos. k-ca. lach. laur. merc. mezer. mosch. nit-ac. nx-m. nx-v. puls. ran-b. rhod. stann. staph. stram.
— — Compare with Vertigo.
**Sensitive**, over-sensitive, etc.: *acon*. alum. am-c. anac. ang. ant-cr. *arn*. ars. asaf. *asar*. *aur*. bar. *bell*. bov. bry. calc. calc-p. camph. cann-s. canth. carb-an. carb-v. caus. *cham*. chin. cic. cina. clem. cocc. *coff*. colch. coloc. con. creos. crotal. cupr. daph. dig. dros. ferr. gels. hepar. hyos. *ign*. *iod*. k-ca. lach. laur. *lyc*. mag-m. *marum*. meph. *merc*. mezer. nat-c. nat-m. nat-s. nitr. *nx-v*. phos. ph-ac. plat. psor. *puls*. ran-b. sabad. samb. sars. *seneg*. *sep*. spig. stann. staph. *sulph*. tabac. *valer*. verat. viol-od. znc.

— Compare with Intolerance, etc.
— to **agreeable** impressions: arn.
— — **ailments**, the most trifling: nx-v.
— — **anxious**: ars.
— — **bright** light: acon. ars. colch. nx-v.
— — **conversation**: *ars.* aur. con. nx-v. znc.
— — **crying** of children: phos.
— — **disagreeable**: arn. sulph.
— — **external** impressions, to all: arn. clem. *cocc.* iod. *nx-v.*
— — **jests**: ang. spig. viol-tr.
— — **light**: nx-v.
— — **looked** at or touched: *ant-cr.* ant-t. ars. cham. cina.
— — **mental** impressions: phos. znc.
— — **moral** impressions. all-s. dig. *ign.* psor.
— — **music**: acon. caus. cham. merc. nat-s. nx-v. sabin. viol-od.
— — **noise**: alum. aur. bar. calc. caus. k-ca. mang. nx-v. sep. sil. znc.
— — **odors**: *colch.* nx-v. phos. sulph.
— — **pain**: *acon.* agar. arn. *ars. aur.* bar. bell. canth. *cham.* chin. cina. cocc. *coff.* colch. con. cupr. *lyc.* mag-c. nat-c. *nx-v.* petr. *phos. sep.* spig.
— — **reading**: crotal. lach. merc.
— — **rudeness**: colch.
— — **scratching** on linen: *asar.*
— — **sensual** impressions: am-c. ars. aur. bar. calc. *chin.* dig. graph. hepar. iod. lyc. mag-c. nat-c. nit-ac. phos. sep. sil. znc.
— — **steel** points directed toward her: apis.
— — **step** and jar on floor, to every: *bell.* nit-ac. nx-v.
— — **talk** to. See Talk.
— — **touch**: *acon. agar.* ant-cr. *ant-t. bell. bry.* camph. cina. cocc. colch. mezer. nx-v.
— — **want** of sensitiveness: bell. chin. con. cupr. cyc. daph. euphr. phos. ph-ac. ran-b. rheum. rhod. sabad. sabin. staph. stram.
— — **wind**, to the: *cham.* sulph.
**Sentimental**: acon. *ant-cr.* calc. cast. coff. *ign.* lach. psor.
**Serene**. See Tranquillity.
**Serious**: *alum.* ambr. am-c. am-m. anac. ang. ant-cr. arg. ars. aur. bar. bell. bov. calc. cann-s. caus. cham. chin. cina. cocc. coff. con. cyc. euph. grat. ign. iod. led. lyc. merc. naja. nat-c. nx-m. olnd. op. ph-ac. plat. plb. puls. rhus. seneg. spig. staph. sulph. sul-ac. thu. verat.
— See also Earnestness, Sad, etc.
— evening: senec.
— when seeing ludicrous things: anac.
**Sexual**, aversion to coition: agn. graph. k-ca. *phos.*
— — — — after menses: berb. *caus.* k-ca. nat-m. *phos.* sep. sul-ac.
— — to men: dios. nat-c. nat-m. puls. raph. stann. sulph.
— — to women: am-c. bapt. dios. nat-m. puls. raph.
— — religious dread of the opposite sex: lyc. puls. sulph.
**Shame**, after an emission: pip-m. staph.
— **want** of (shameless): hell. *hyos.* mosch. nx-v. op. *phos.* phyt. stram. verat.
— — — during lying-in: verat.
— — — exposes the person: HYOS. *phos.* phyt.
**Shrieking** (crying out, etc.): acon. *apis.* arn. ars. arum-t. aur. *bell.* borax. bry. calc. camph. canth. carb-an. *caus.* cham. chin. cic. cina. cocc. coff. croc. cupr. hyos. *ign.* ipec. k-bi. k-ca. laur. lyc. mag-c. merc. nit-ac. nx-v. plat. puls. ran-sc. rheum. seneg. sep. sil. stram. sulph. tanac. verat.
— Compare with Crying, Weeping, etc.
— for aid: plat.
— on going into spasms: verat-v.
— during sleep: cina. ign.
— during cramps in abdomen: cupr.
— in children: *apis.* bell. borax. *cham. cina.* coff. hell. ipec. *jalap. rheum. senn.* stram.
**Shy**. See Bashful.
**Sick**, desires to show that he is: tarent.
— See under Anxiety; also Sick, Well, etc., under Delusions.
**Sighing**: ailan. alum. am-c. arg-n. *bry.* cham. cimic. colch. dig. hell. hura. *ign.* lach. nat-c. op. plb. puls. rhus. sulph. tabac.
— See also under Respiration.
— during fever: ign. puls.
— involuntary: calc-p.
— menses, before: *ign.* lyc.
— — during: ars. cimic. cocc. graph. ign. plat.
— — after: stram.
**Silent**: acon. *agar.* alum. ambr. am-c. am-m. anac. ant-cr. *arg.* arn. *ars.* bar. *bell.* berb. bism. borax. bov. brom. bry. cact. calc. calc-p. cann-i. canth.

caps. *carb-an.* caus. cham. chin. cic. cina. clem. cocc. coff. coloc. con. cupr. cyc. dig. euph. euphr. graph. grat. guai. *hell.* hepar. hipp. hydr. *hyos.* ign. ipec. k-bi. k-ca. lach. led. lil-t. *lyc.* mag-c. mag-m. mag-s. manc. *mang.* men. merc. mezer. murx. mur-ac. nat-c. nat-m. nat-s. nicc. nit-ac. nx-j. nx-m. nx-v. olnd. op. ox-ac. petr. phos. *ph-ac. plat.* plb. *puls.* rheum. sabad. sabin. sars. sep. sil. spong. squil. stann. staph. stront. sulph. sul-ac. tabac. tarax. tarent. thea. thu. tong. verat. viol-od. viol- tr. *znc.*
— Compare with Answer, does not.
— in open air: plat.
— afternoon: hell.
— after depression: ziz.
— after eating: arg-n. ferr-mag.
— during headache: con.
— during menses: cast. mur-ac.
— except in delirium or to find fault: verat.

**Silly**. See Foolish.

**Singing**: *bell.* cann-i. cann-s. caps. *cic.* cocc. *croc.* cupr. hipp. hydr. hyos. lyc. mag-c. manc. *marum.* merl. mezer. nat-c. nat-m. op. phos. plat. sep. *spong. stram.* tabac. tarent. therid. verat.
— **alternating** with anger: croc.
— — — distraction: spong.
— — — groaning: bell.
— — — hatred of work: spong.
— — — vexation: agar. croc.
— — — weeping: acon. bell. stram.
— **amorous** songs: hyos.
— **involuntarily**: *croc.* hydrph. marum.
— **joyously**, at night: verat.
— **obscene** songs: alco. hyos. stram.
— **in sleep**: bell. croc. ph-ac.

**Sits** quite stiff: cham. hyos. puls. sep. stram.
— still: cham. puls.
— as if wrapped in deep, sad thoughts, and notices nothing: cocc.
— in one place for three or four days, during headache: con.
— with head on hands and elbows on knees: glon.

**Size**, incorrect judge of: stram.
— of frame seems lessened: phys.
— See under Delusions, Distance, etc.

**Slander**, disposition to: am-c. anac. ars. bell. borax. hyos. ipec. lyc. nit-ac. *nx-v.* petr. sep. stram. verat.

**Slowness**: asar. *puls.*
— of purpose: graph.

**Sluggishness**. See under Mind and Thoughts.

**Smaller**, things appear: plat. stram.
— Compare Distance, Size, etc., and see under Delusions.
— seems to be getting: calc.

**Smiling**, foolish: bell. merc.
— involuntarily: aur. bell. lyc.
— — when speaking: aur.
— never: alum.
— sardonic: bell.
— in sleep: cadm.

**Sneers** at every one: alum.

**Sobbing**: alcoh. ars. caus. cocc. hell. led. lobel. lyc. nx-v. phos. sep. stram. tarent.
— Compare Sighing and Weeping.
— convulsive: strych.
— horizontal position, on assuming: euphr.
— lonesome condition, over his: lith.
— menses, after: stram.
— paroxysmal: phos.
— in sleep: alum. ipec. nat-m. *op.*
— after vexation: *znc.*
— violent: hydr-ac.
— on waking: carb-an. cina.
— from worry: lyc.

**Solemn**. See Serious.

**Solicitude**. See Anxiety.

**Solitude**, See Company, averse to.

**Somnambulism**: acon. agar. alum. bry. cic. hyos. kalm. mosch. nat-m. op. petr. *phos.* rheum. rumx. sil. stann. sulph. verat. znc.

**Sorrowful**. See Sadness.

**Speech**, abusive: bell. stram.
— **anxious**, in sleep: alum. graph. nx-v. sulph.
— **babbling**: plb.
— **business**, of: bry. hyos. sulph.
— — in sleep: com. rhus. sulph.
— **calculations**, makes aloud in dreams: selen.
— **changeable** from one subject to another, quickly: agar. cimic. ether. *lach.* par.
— **childish**: acon. arg-n.
— **confused**: alcoh. bell. calc. caus. crotal. *hyos.* lyc. mosch. stram.
— — in sleep; *colc.*
— **delirious**: *bell.* bry. camph. canth. coff. cup-ac. dig. *hyos. lyc.* op. phos. plb. rheum. sil. sulph. tabac. tilia. vip.

— — Compare with Delirium.
— — at night: cact. dig. rheum. sil.
— — during chill: cham.
— — — — on being aroused; hepar.
— — during heat: coff. tilia.
— — menses, before: lyc.
— — in sleep: bell. rheum.
— — — on falling asleep: cact. phos.
— — on waking: bry.
— **disconnected**: bell. coca. cupr. hydr-ac. hyos. merc. phos. plb. sal-ac. stram.
— — on waking: *cact.* ign. op.
— **dreams**, about: znc.
— **drunken**, as if: hyos. lyc. meph.
— **embarrassed**: atrop. merc. pallad.
— **everything**, about: hyos. op.
— **excited**: morph. nat-c.
— **extravagant**: ether. plb. stram.
— **faults** of others: ars. verat.
— **fine**: hyos.
— **foolish**: bell. phos. par. *stram.* tabac.
— **foreign** tongue, in a: *stram.*
— **future**, about: hyos.
— **hasty**: acon. alcoh. ambr. ars. *bell.* bry. camph. cann-i. cann-s. cimic. cina. cocc. hepar. hyos. lach. *merc.* mosch. nx-v. ph-ac. plb. stram. thu. verat.
— **health**, about his: nx-v.
— **hesitating**: absin. k-bro. merc. morph. vip.
— **himself**, to: ant-t. hyos. k-bi. mosch. nx-m. plb. rhus. vip.
— **incoherent**: absin. *agar.* alcoh. *arg-n.* ars. bell. calad. camph. CANN-I. cub. cupr. dulc. ether. hepar. hyos. k-bro. lach. morph. op. plb. rhus. *stram.* tanac. vip.
— — night: plb.
— — during sleep: k-bi. phos.
— — after dozing: op.
— **intend**, says what she does not: nat-m.
— **interchanges** words: am-cau. k-bro.
— **little**: agar. ars. mang. nat-s. *ph-ac.* staph.
— — Compare Silent.
— **loud**: nx-m.
— **merry**: agar. ether.
— — in sleep: mur-ac.
— **mistakes**, makes. See Mistakes, makes.
— **monosyllabic**: ars. merc. nx-v. *ph-ac. sul-ac. thu.*
— **nonsense**: acon. aur. *bell.* cann-i. canth. chlorof. ether. *hyos.* k-bi. merc. *stram.* sulph.
— — night, in sleep: cact.
— — on springing up while asleep: k-ca.
— — in reverie: aur.
— **obscene**: aur. bell. *stram.*
— **prattling**: aloe. *bry.* calad. *hyos.* nx-v. stram. tarax.
— — in sleep: nx-v.
— **random**, at: at night: plb.
— **rapid**. See Hasty.
— **religious**: stram. *verat.*
— **science**, of: cann-i.
— **strange**: cham. ether. gall-ac.
— **subject**, cannot free himself from the one: petr.
— **thought**, expresses in sleep what she thought when awake: am-c.
— **threatening**: tarent.
— **truth**, never tells the: verat.
— **unintelligible**: acon. ars. bell. calen. euph. *hyos.* lyc. *merc.* naja. nx-v. secale. sil. *stram.* tabac.
— — in sleep: arn. atrop. cast. cham. mur-ac.
— **vexations**, about old: cham.
— **vivacious**, animated: cann-i. *hyos.*
— **violent**: bell.
— **wandering**: acon. æth. arn. ars. aur. *bell. bry.* calc. *camph.* canth. cham. chin. cic. cina. coloc. cupr. dulc. *hyos.* ign. k-ca. *lach. lyc.* merc. nx-m. nx-v. *op.* phos. *plat.* plb. puls. rheum. rhus. sabin. secale. spong. *stram.* sulph. *verat.*
— — at night: aur. bell. bry. coloc. dig. op. rheum. sep. sulph.
— **war**, of: bell. hyos.
— **wild**: atrop. camph. hyper. lyc. plb.
— **witty**: caps. thea.

**Spelling**, difficult: lach.
— mistakes in. See Mistakes.

**Spinning**, imitates: hyos.
— around on one foot: cann-s.

**Spoken** to, averse to being: ars. cham. gels. nat-m. nat-s. nx-v. rhus. staph.
— — cannot bear the talk of others: agar. *am-c.* ARS. con. mag-m. marum. *znc.*
— — wants to be let alone: hell. helon.
— — See also Talk.

**Starting**: acon. alum. ang. ant-cr. arn. ars. bell. *borax.* calc. cann-s. carb-an. caus. cham. cic. cocc. con. graph. hyos. *ign.* k-ca. lac-can. lach. led. lil-t. lyc. merc. nat-c. nat-m. *nit-ac.*

*nx-v. op.* petr. phos. plat. rhus. sabad. *samb.* sep. *sil.* spong. *stram. sulph.* sul-ac. therid. verat.
— Compare with Limbs, Muscles, etc., under Extremities.
— **anxious**: aloe. apis. lyc. *sulph.*
— — from downward motion: *borax.*
— **convulsive**: ars. calc-p. hyos. strych.
— **easily**: ant-t. borax. calc. *camph.* k-ca. merc. nat-m. nat-p. nit-ac. nx-m. nx-v. op. phos. phys. *sep. sil.* tabac. *therid.* verat. znc.
— **electric**, as if: cann-s. euph.
— — wakening her: arg.
— **falling**, as if: bell. bism. mezer.
— **feet**, as if coming from the: lyc.
— **fright**, from: *acon. am-c.* anac. apis. arn. BELL. bry. calc-p. carb-v. *caus.* coff. creos. dig. HYOS. hyper. lyc. mag-s. *merc.* merc-c. mosch. nat-ars. nat-c. op. plb. sabad. sars. sil. staph. STRAM. stront. *sulph.* verat.
— **paroxysmal**: ars. rhus.
— **suffocated**, as if: aur-m.
— **tremulous**: cham.
— **twitching**: con.
— **unconscious**, in sleep: znc.

**Conditions** of Starting.
— **morning**, from sleep: chin-s. clem. sabad. spong.
— **evening**, during heat: puls.
— — on falling asleep: ambr. am-m. arn. *ars.* bar. *bell.* dulc. k-bi. merc-c. nat-c. sars. stront. *sulph.*
— — jerking or twitching, ceasing on falling asleep: *agar.* hell.
— — in sleep, a: calc. k-iod.
— **night**: carb-v. euph. indg. lyc. mag-c. merc-c. morph. sil. staph. stram. sulph.
— **awake**, while lying: anac. bry. euph.
— — awakening, see under Sleep.
— **bed**, in: cic. hura. tabac.
— **dreams** in or during: ant-cr. petr. *puls. sulph.*
— — from: coral. nat-c.
— **lying**, when: lyc.
— — on back: calc-p. mag-c.
— **menses**, during: znc.
— **noise**, at any: ant-cr. apis. bar-ac. carb-v. k-iod. nat-c. nat-p. *op.* rhus. sabad. sil.
— — when a door is opened: hura. merc. mosch. phos.
— — — — — slams: oxal-ac.
— — fall, on hearing anything: alum.
— **pain**, during: sumb.
— — in stomach: nicc.
— — a tearing in the wrist: merl.
— **palpitation**, from: dig.
— **prick**, of a needle, at the: calc.
— **sleep**, before: alum.
— — **on falling**: ambr. am-m. arn. *ars.* bar. BELL. carb-an. carb-v. chin. coff. dulc. k-bi. *lyc.* mag-c. merc. merc-c. nat-c. nat-m. nat-s. *nit-ac.* op. pæon. phos. plb. sars. sep. stront. strych. *sulph.* tabac.
— — **during** sleep: agn. alum. apis. arn. *ars.* atrop. bell. brom. bry. calad. calc. calc-p. camph. canth. cast. *caus. cham.* creos. cyc. iod. *ipec.* iris. graph. hura. *hyos.* hyper. *k-ca.* k-iod. laur. lyc. mag-c. mag-m. merc. merc-c. mezer. morph. nat-m. *nx-v.* op. oxal-ac. petr. phos. puls. sars. seneg. sep. sil. spig. stann. stram. *sulph.* thu.
— — **from** sleep, awakening: *acon.* alcoh. ant-t. apis. ars. aur m. BELL. benz-ac. caps. chel. chin. chin-s. cinnb. clem. cocc. coff. conv-d. cyc. *dig.* dros. ferr-iod. gins. k-iod. led. lup. lyc. mag-c. *merc-c.* murx. mur-ac. nat-c. nit-ac. nx-v. plb. ruta. sabad. *samb. sil.* spong. stram. *sulph.* sul-ac. tarent. thea. znc.
— — — as if suffocated: *samb.*
— **tossing** of arms, from: merc.
— **touched**, when: k-ca.
— **trifles**, at: arn. calc. cham. cocc. hura. *nat-m.* nx-m. nx-v. sabad. *sil.* spong. sul-ac. znc-m.
— **uneasiness**, from: mur-ac.
— on **waking**: pallad.

**Stories**, exciting, aggr.: calc. marum.

**Stranger**, sensation as if one were a: valer.
— presence of, aggr.: *ambr.* bar. lyc. petr. *sep. stram.*
— See also under Delusions.

**Striking**: *bell.* canth. elaps. hydr. *hyos.* lil-t. lyc. mosch. nat-c. phos. *stram.* stront. *verat.*
— in children: cina.
— about him: *bell.* hyos. lyc. phos. stram.
— desires to strike: bell. elaps. hydr. lil-t. nat-c.
— his face: bell.
— his head: ars. tarent.
— himself: tarent.

**Stubborn.** See Obstinate.
**Study,** aversion to: æsc-h. carb-an. coca. coloc. gels. ham. hipp. hydr. kalm. k-bi. phos. pic-ac. plan. sol-n.
— desire to: cob. gels. nat-p.
— is difficult: nat-p. sep.
— impossible: cop. dig. ferr. glon. gymn. hyos. ham. kalm. lyc. morph. nx-v. pic-ac. ptel. sumb.
**Stupefaction,** See under Head.
— also compare Stupidity.
**Stupidity,** dull, sluggish mind, etc.: acon. æsc-h. alum. ambr. anac. apis. arg. asar. aur. bar. bell. bov. bry. bufo. calc. calc-p. cann-i. canth. caps. carb-an. caus. chel. chin. chro-ac. *cic.* cocc. coc-c. corn. creos. croc. crotal. cupr. dig. dios. dros. dulc. ether. gels. hell. hepar. *hyos.* ign. indg. k-ca. k-sul. *lach.* laur. led. lil-t. mag-m. marum. merc. mezer. mosch. mur-ac. naja. nat-ars. nat-c. nx-m. nx-v. olnd. op. par. phos. ph-ac. phys. pip-m. puls. rheum. rhod. rhus. rhus-v. ruta. sal-ac. sang. sars. secale. sil. spong. still. stram. sulph. sul-ac. tarax. tarent. tilia. valer. verb. znc.
— Compare with Confusion, Thoughts, etc.; also with Stupefaction, etc., under Head.
**Conditions** of Stupidity.
— **morning**: agar. bar. carb-an. k-ca. nat-ars. *ph-ac.* phys.
— **afternoon**: atrop. dios. ham. pip-m. plan.
— **evening**: lyc. pip-m. sep.
— **night**, on waking: phos. plat. verat.
— **beer**, after: coloc.
— **dinner**, aggr. after: carb-an.
— **fever**, after: sep.
— **headache**, from: dios. dulc. glon. plat.
— **menses**, during: lyc.
— rising from bed, on: oxal-ac.
— **sleep**, after sound: mezer.
— **smoking**, from: acon.
— **vertigo**, during: hell.
— **waking**, on: anac. bar. clem. op. phos. plat. verat.
— **walking**, on: phys.
— — from pressure in forehead: *ph-ac.*
— — in open air, amel.: plan.
— **warm** room, on entering: acon.
— **wine**, from: acon.
**Stupor.** See Unconsciousness.
**Stuttering.** See under Speech.
**Succeeds,** never: am-c. nat-s.
— believes everything will fail: arg-n.
**Suggestions,** will not receive: helon.
**Suicide,** disposition to commit: alum. ambr. am-c. ant-cr. ant-t. arg-n. *ars.* AUR. aur-m. bell. caps. carb-v. caus. chin. cimic. clem. creos. crotal. dros. grat. *hepar.* hipp. hyos. lach. merc. mezer. *nat-s.* nit-ac. *nx-v.* plb. *puls.* rhus. secale. sep. sil. spig. stram.
— — with dread of an open window or a knife: chin.
— — seeing blood on a knife, she has horrid thoughts of killing herself, though she abhors the idea: alum.
— — by **drowning**: ant-cr. bell. dros. hell. hyos. puls. rhus. secale. sil. verat.
— — by **hanging**: ars. bell.
— — by **poison**: lil-t.
— — by **shooting**: ant-cr. aur. carb-v. hepar. nat-s. nx-v. puls.
— — by **throwing** himself from a height: aur. bell. crotal. nx-v. stram.
— **conditions** of Suicide.
— at **night**: ant-cr. chin. nx-v.
— — in twilight: rhus.
— **evening**: chin. dros.
— — during sadness: hepar.
— **fear** of death, during: tabac.
— **fever,** intermittent, during: *ars.* bell. chin. lach. *spong.* stram. valer.
— — during the sweat: alum. *ars. aur.* CALC. hepar. *merc.* sil. *spong.*
— **menses,** during: sil.
— **palpitation,** during: aur. nx-v.
— **walking** in open air, when: bell.
**Superstitious** ideas: con.
**Suspicious:** acon. ambr. *anac.* ant-cr. aur. *bar. bell.* calc. cann-i. canth. *caus.* cham. *cic.* cimic. con. crotal. cupr. dig. dros. hell. hyos. k-bi. *lach.* lyc. merc. nat-c. nit-ac. nx-v. op. phos. plb. *puls.* rhus. ruta. selen. stann. staph. sul-ac. verat-v. viol-tr.
— of the future: anac. caus.
**Swearing.** See Cursing.
**Sympathy,** excessive: caus. iod. lyc. nat-c. nat-m.
**Taciturn.** See Silent.
**Talk** to some one, desires to: lil-t.
— is slow to learn to: nat-m. nx-m.
— of others aggr.: am-c. *ars.* chin. colch. con. mag-m. mang. *nat-c. nx-v.* rhus. *sep.* sil. verat. *znc.*
— of unpleasant things aggr.: *calc.* cic. ign. *marum.*

**Talkative.** See Loquacious.
**Talking.** See Speech.
— averse to. See Silent.
— Compare with Speaking.
**Tears** things: *bell.* camph. hyos. op. *stram.* sulph. verat.
— pillow with teeth : phos.
— his own person : ars.
**Tender** mood: croc. *ign.* manc. nx-v. phos.
**Thinking** of complaints, aggr.: alum. *bar. calc.* dros. *hell.* helon. lach. *nit-ac.* olnd. *oxal-ac.* plb. *ran-b.* sabad. spig. spong. staph.
— — — ameliorates: *camph.* cic. *hell,* mag-c. prun.
**Thoughts**, alienation of. See Insanity.
— **clearness** of; ability to think, etc.: acon. alum. ambr. anac. ang. ars. asar. aur. bell. bov. calc-p. cann-s. carb-v. cham. chin-s. cimic. coca. coc-c. coff. creos. ether. ham. hell. ign. k-bro. k-ca. lach. lact-ac. laur. merc. mezer. nat-m. nit-ac. olnd. op. *phos.* pip-m. rhus. sep. spig. spong. *thea.* thu. verat. viol-od.
— **concentration** of, difficult: brom. ham. hell. lil-t. lyc. merl. ox-ac. phys. sars. tabac.
— — — impossible: acon. ailan. alum. apis. bapt. cact. cinnb. corn. croc. dros. gels. hell. hydr-ac. iris. laur. lil-t. lyc. merc. nat-c. nat-m. ox-ac. ph-ac. phys. ran-sc. sang. selen. senec. sep. sil. spig. thu.
— **confusion** of: absin. acet-ac. *acon.* æsc-h. æth. agar. ailan. alcoh. am-c. am-m. anac. ang. ant-t. apis. apoc. arg-n. *arn.* ars. asaf. atrop. aur. bapt. *bell.* benz-ac. berb. bism. borax. bov. bry. calc. camph. cann-s. carb-an. caus. canth. carb-v. cham. *chin.* chin-s. chro-ac. cic. cina. cinnb. clem. coff. coloc. con. cop. corn. creos. croc. cupr. cup-ars. cyc. dig. dulc. ethn. fluor-ac. gels. gins. *glon.* gran. grat. *hyos.* hydr-ac. *hyper.* ign. iod. jabor. k-bro. k-ca. lach. lact-ac. lact. laur. lil-t. led. *lyc.* merc. mezer. morph. mosch. murx. naja. nat-ars. nat-c. nat-m. *nx-m. nx-v.* olnd. *op.* phos. *ph-ac.* plan. plb. ptel. puls. ran-b. raph. rhus. ruta. sabin. sal-ac. samb. sang. secale. seneg. *sil.* spig. stann. staph. *stram.* strych. sulph. syphil. tabac. tanac. trom. valer verat. verb. viol-od. vip. znc.
— — **Compare** with Mistakes; also with Confusion under Head.
— — of present with past: cic.
— — of present with future: anac.
— **Conditions** of Confusion of Thoughts.
— — **morning**: agar. chel. mag-s. phys. sep. strych. sulph. sumb.
— — — on rising, amel.: mag-s.
— — — on waking: agar. mag-s.
— — **afternoon**: cann-s.
— — **evening**: calc-s. cann-s. coloc. dios. nat-c.
— — **night**: cedr. corn. mur-ac. secale.
— — — on waking: chel. mezer. plat.
— — **beer**, from: coloc.
— — **conversation** of others, from: calc. nat-m.
— — **coffee** or wine, from : all-c.
— — **eating**, after: sil. tabac.
— — **excitement**, amel.: chin. cyc.
— — **eyes**, on closing: atrop.
— — **mental** labor, from: mag-c. sulph.
— — **pain** in vertex, with: glon.
— — **reading**, when: apis. lyc.
— — **remarks** of others, from : *mezer.*
— — **rising** from couch, on: bell.
— — **smoking**, on: petr.
— — **standing** still, amel.: iris-f.
— — **vertigo**, during: ars. sulph.
— — **vomiting**, amel.: tabac.
— — **waking**, on: agar. grat. mag-s. staph. sulph.
— — **will**, strong effort of, amel.: glon.
— **connection** of, difficult: am-c. asaf. borax. caps. *chin.* lact. *nat-c.* nx-v. ph-ac. sulph. verat. znc.
— — lost: alum. arg-n. lyc. nx-v. sulph.
— — can't read or calculate: nx-v.
— of **death**: am-c. carb-an. caus. clem. con. crot-c. hura. tarent. znc.
— — would die: ACON. agn. *apis.* cimic. creos. dig. hell. hepar. merc. rhus. sul-ac. tabac.
— — — Compare with Anxiety, Fear, etc.
— **deep**: bell. mur-ac. grat.
— — about his future: spig.
— **deficiency** of: acet-ac. *acon.* agn. alum. *am-c.* anac. arg-n. asaf. asar. atro. aur. bell. bov. calc. calc-p. camph. cann-s. canth. *caus.* cham. chin. cic. clem. cocc. coff. corn. creos.

croc. cupr. cyc. dig. glon. guai. hell. hyos. ign. iod. ipec. k-bro. *lach.* lil-t. lyc. men. merc. merc-c. *mezer.* nat-c. *nat-m.* nit-ac. nx-m. olnd. op. petr. *ph-ac.* plb. rhus. sep. sil. staph. sulph. tarent. thu. valer. verat. *znc.*
— — on extra exertion: olnd.
— — from any interruption: colch.
— — vomiting, amel.: asar.
— **difficulty** of thinking or comprehending, etc.: *alum.* ambr. am-c. arg-n. aur. bell. berb. bry. *calc.* calc-p. camph. carb-v. cham. chel. con. creos. croton. *dig.* gins. *hell.* hydr-ac. *hyos.* ign. iod. lach. lact. lyc. men. merl. mezer. nat-c. *nat-m.* nit-ac. nx-m. *nx-v.* *olnd.* *op.* petr. ph-ac. phys. plan. plb. rhus. sabad. secale. sep. *sil.* *spig.* *staph.* stram. sulph. therid. thu. viol-od. *znc.*
— — Compare with Concentration.
— — with chronic dullness of mind, and with sensation as if a board were pressing against the head: sulph.
— — unable to think long or much: cham. cinnb. ph-ac. pic-ac. stram.
— — could not think of her condition: chel.
— **Conditions** of Difficulty of Thinking.
— — **morning**: anac. borax. canth. guai. nat-c. ox-ac. phos. *ph-ac.* sil. sulph.
— — — on waking: anac. stann.
— — — on rising: stram.
— — **afternoon**: all-c. anac. ang. cann-s. hyos. laur. lil-t. nat-m. rhus-r. sep. sil.
— — **evening**: anac. cann-s. dig. hipp. ign. lach. lyc. mill. nat-m. rhus.
— — **air**, in open: plat.
— — — amel.: cinnb. men.
— — **alone**, when: ph-ac.
— — **company**, in: plat.
— — **eyes**, on shutting the: znc.
— — — — amel. on: k-ca.
— — **food**, after: calc-s. graph. rhus. tabac.
— — — amel.: sil.
— — **headache**, with: am-c. apis. ars. bell. cann-i. cann-s. carb-v. cupr. lept. lyc. mezer. mosch. phos. ptel. rhus. stram. viol-od.
— — — confusion of head, with: anac. arg-n. bov. calc. con. cop. dig. ferr. hæmat. iod. lach. lyc. men. merc. nit-ac. nitr. op. phos. sulph. tabac. thu.
— — — fullness of head, with: arg-n. borax.
— — — heat of head, with: agar. arg-n. dig.
— — — heaviness of head, with: arg-n. calc. gels. hæmat. hell. tabac.
— — **lying**, when: bry.
— — — amel.: znc.
— — **moving**, from: rhus.
— — **menses**, during: calc.
— — **mental** exertion, from: hepar. hura. ign. lyc. nx-v. ran-b. sulph.
— — **news**, from disagreeable: calc-p.
— — **reading**, on: acon. agn. alum. ang. coff. con. glon. hipp. lach. mezer. nx-m. nx-v. olnd. ph-ac.
— — **room**, in a: men.
— — speaking, on: am-c. k-ca. mezer.
— — **vertigo**, with: ambr. arg. ars. borax. bry. calc. camph. canth. dig. gels. hæmat. merc. nat-c. phos. ph-ac. rhus. sil. stann. stram. sulph.
— — **vomiting**, amel.: asar. tabac.
— — **waking**, on: anac. con. stann.
— — **wine**, after: all-c. mill.
— — **writing**, when: acon. cann-s. chin-s. glon. mag c. nx-m. rhus.
— **disconnected**: cann-i. *cupr*-ac. dig. gels. nx-v. rhus-v. viol-od. znc.
— — on attempting to fix them has a vacant feeling: gels.
— — only in fragments, in following an idea: nat-m.
— — when calculating or reading: *nx-v.*
— — when talking: merc-c.
— **evil**: lach.
— **expression** of is difficult. See under Speech.
— **facetious**: nx-m.
— **fixed**. See Persistent and compare with Delusions.
— **flow** of; ideas are abundant, etc.: agar. alcoh. alum. ambr. am-c. anac. ang. *ars.* aur. *bell.* borax. *bry.* *calc.* cann-i. cann-s. canth. caus. *chin.* cob. coca. cocc. *coff.* coloc. eupion. glon. graph. hepar. hyos. k-ca. lach. *lyc.* merc. morph. mur-ac. nit-ac. nitr. *op.* *phos.* ph-ac. plat. *puls.* rhus. sabad. sep. sil. spong. staph. stram. *sulph.* tabac. tereb. thea. valer. verb. *viol-od.* viol-tr. znc.
— — **evening**, before going to sleep:

*chin.* lyc. *nx-v.* *puls.* sabad. sil. *staph.* viol-tr.
— — **night**, at: aloe. borax. *calc.* chin. coca. *coff.* colch. graph. hepar. k-ca. *lyc.* *nx-v.* pic-ac. puls. sabad. sep. sil. *staph.* sulph. tabac. viol-tr.
— — in partial sleep: hyos.
— **frightful**: phos. phys.
— — on seeing blood on knives: alum.
— **future**, of the: cyc. senec. sep.
— **impaired**. See Deficient, Loss of: etc.
— **inability** to think. See Difficulty, under Thoughts.
— **loss** of, ideas, etc.: abrot. acon. æth. agar. ambr. anac. apis. ars. bell. brom. calc. calc-p. cann-s. canth. caus. cham. chel. chin. *cic.* cinnb. clem. cocc. coff. coloc. *con.* corn. creos. croc. cupr. cyc. dig. elaps. ether. ferr. *gels.* glon. graph. guai. gymn. hell. hepar. hipp. hydrph. hydr ac. hyos. ign. ipec. k-bi. k-bro. k-iod. kalm. lach. laur. led. lil-t. lyc. mag-c. manc. merc. mezer. morph. mosch. nat-ars. *nat-m.* nit-ac. ol-an. op. petr. *phos.* *ph-ac.* pic-ac. plan. plb. ptel. ran-b. raph. rhod. rhus. ruta. secale. selen. sep. sil. stann. stram. tabac. thu. trom. verat-v. viol-od. znc.
— — as if in a dream: bell. cham. ol-an. squill. znc.
— — as in a reverie: arn. chin. grat. guai. ign. mezer. ol-an. rhus. spig. sulph.
— **Conditions** of Loss of Thoughts.
— — **morning**: anac. phos. ph-ac. stram. thu.
— — **afternoon**: graph. sep.
— — **evening**: laur. lyc. naja. nat-m. rhus. sep.
— — **night**: chin. sil. sulph.
— — **exertion**, from: nat-m.
— — **fever**, during: sep.
— — **food**, after: ferr. mag-c. nat-m. rhus.
— — — amel., evening: sil.
— — **headache**, during: apis. calc. glon.
— — **menses**, during: raph.
— — **motion**, on: laur.
— — **reading**, when: corn. hyos. lyc. merc. *op.*
— — **standing**, when, at breakfast time: guai.
— — **vertigo**, from: ran-b.
— — **waking**, on: chin. k-bro. k-ca. ptel. sil. thu.
— — — amel., when spoken to: ol-an.
— — **walking**, when, after eating: rhus.
— **past**, of the: cann-i. men. nat-m. senec.
— **persistent**: æth. *cann-i.* carb-v. cham. graph. *ign.* iod. nx-m. petr. ph-ac. phys. plat. puls. sulph. tarent. thea. thu.
— — See also under Delusions.
— — of ideas which first appeared in his dreams: psor.
— — about a garment made the previous day: æth.
— — about music: ign.
— — thinks mind and body are separated: anac. thu.
— **rapid**, quick, etc.: acon. æsc-h. alcoh. ang. cann-i. caus. cob. *coff.* hyos. kalm. lach. op. ox-ac. sabad. valer. verat. viol-od.
— **reflect**, or think, desires to: aur. chin. eugen. ham.
— **repetition** of: stram.
— — — words and sentences: sulph.
— **slowness** of, sluggishness of, etc.: acon. æth. *alum.* ambr. am-c. arg-n. aur. bell. *calc.* carb-v. caus. *chin.* con. cyc. dig. graph. *hell.* *hyos.* ign. iod. ipec. k-bro. *lach.* *lyc.* men. merc. nat-c. *nat-m.* nit-ac. *nx-m.* *nx-v.* olnd. *op.* ox-ac. petr. *phos.* ph-ac. *plb.* rhus. ruta. sabad. sars. sep. sil. *staph.* stram. sulph. *thu.* verat.
— **thoughtful**: acon. am-m. arn. bar. bell. brom. calc. cann-s. canth. *carb-an.* cham. chin. cic. clem. *cocc.* cyc. euph. euphr. grat. hyos. *ign.* ipec. *lach.* lyc. mag-m. manc. mang. mezer. nat-c. nit-ac. nx-v. *phos.* plb. ran-b. rhus. sabad. *sep.* spig. *staph.* stront. sulph. thea. thu. tilia. viol-od.
— **thoughtless**: agn. alum. ambr. am-c. am-m. anac. asaf. bell. bov. cann-s. canth. caus. cham. cic. clem. coff. creos. croc. cupr. daph. evon. guai. hell. hepar. hyos. ign. k-ca. lach. lyc. merc. mezer. nat-c. nat-m. nit-ac. nx-m. olnd. phos. ph-ac. rhod. rhus. ruta. sep. spig. valer. verat. znc.
— — from the least thing which affects her: croc.
— **unpleasant** subjects, of: *benz-ac.* *cocc.* *chin.* men. mezer. nat-m. rhus. sep.
— — — at night: chin. *rhus.*
— **vanishing** of: anac. apis. asar.

borax. bry. calc. *camph.* *cann-s.* canth. carb-an. cham. coff. creos. cupr. evon. gels. guai. hell. hepar. iod. k-ca. *lach.* merc. mezer. nit-ac. nx-m. nx-v. ol-an. op. plant. puls. *ran-b.* ran-sc. rhod. rhus. staph. viol-od.
— — — **mental** exertion, after: asar. mezer. staph.
— — — **headache**, during: bell.
— — — **reading**, on: bry.
— — — **speaking**, when: mezer. staph.
— — — — when spoken to: sep.
— — — **vertigo**, during: ipec. op.
— — — **writing**, when: rhus.
— **wandering**: *acon.* all-s. alcoh. *aloe.* am-c. ang. apoc. arn. atrop. *bapt.* bell. cann-i. cann-s. caus. cic. colch. coloc. corn. crotal. cupr-ac. dig. ferr. glon. *graph.* ign. iod. k-bro. lach. lyc. manc. merc. naja. nat-c. nat-m. nit-ac. olnd. op. ph-ac. phys. pic-ac. plect. plb. *puls.* staph. tabac. valer. viol-od. *znc.*
— — during menses: calc.
— — when studying: ham. phys.
— — when writing: iris. *nx-m.*
— — when talking: merc-c.
**Throws** things away: dulc. staph. tarent.
— — at persons: bell. [etc.
— — Compare with Tossing, Refuses,
**Time**, fritters away his: cocc. nx-v.
— passes too slowly: alum. arg-n. bar. con. lyc. mag-m. nat-c. nx-v. pallad. petr. plb.
— — a few seconds seem ages: cann-i.
— — See also Ennui.
— passes too quickly: coca. cocc. thea. therid.
**Timidity**: acon. aloe. alum. anac. ang. ars. aur. bar. bell. bry. calc. canth. carb-an. caus. chin. cocc. coff. con. cupr. daph. graph. hyos. ign. iod. ipec. k-ca. laur. lil-t. lyc. mag-c. mur-ac. *nat-c.* nat-m. nit-ac. nitr. op. phos. plat. *puls.* ran-b. rhus. secale. sep. sil. spig. spong. staph. *stram.* sulph. sul-ac. tabac. verat. znc.
— about going to bed: caus.
— alternating with assurance: *alum.*
**Touched**, aversion to being: *ant-cr.* ars. ant-t. cham. cina. thu.
**Tranquillity**: æsc-h. aloe. ars. bell. caus. chin-s. clem. coca. *coff.* croc. dros. ether. eucal. euph. gins. gran. hydr-ac. *hyos.* ipec. k-bro. lach. laur. led. lil-t. lyc. men. merl. mosch. mur-ac. naja. nat-m. op. petr. phos. PH-AC. plat. plb. seneg. sil. spig. stann. staph. verat. viol-tr. znc.
— incomprehensible: morph.
— **mental**: arn. cham. *chel.* chin. *cic.* cyc. ether. ferr. gins. hyos. ign. k-bro. lach. manc. men. mezer. op. plat. spig. tellur.
— — after anger: ipec.
— — morning on waking: chel.
— — except while chest symptoms lasted: chel.
**Travel**, desires to: merc.
**Trifles**, seem important: ferr. ipec.
— with everything: agar.
**Trustful**: hydroc. spig.
**Truth**, never speaks the; does not know herself what she is saying: verat.
— believes all she says is a lie: lac-can.
**Unattractive**, things seem: chin.
**Unconsciousness**: absin. acet-ac. acon. æsc-h. æth. agar. ailan. alum. ambr. am-c. am-m. anac. ant-cr. ant-t. apis. arg-n. *arn.* *ars.* asar. *bapt.* bar. bell. bism. bov. bry. calad. calc. *camph.* cann-s. canth. carb-v. cham. chel. chin. chin-s. *cic.* cimic. *cina.* cocc. *coff.* colch. *con.* creos. croc. *cupr.* cupr-ac. *cyc.* dig. dulc. elaps. ether. euph. ferr. fluor-ac. *gels.* *glon.* graph. guai. *hell.* hepar. *hydr-ac.* *hyos.* ipec. k-bi. k-ca. k-iod. *lach.* *laur.* lact. *led.* *lyc.* mag-m. manc. merc. *merc-c.* mezer. mosch. *mur-ac.* naja. nat-c. nat-m. nit-ac. nitr. *nx-m.* *nx-v.* ol-an. olnd. *op.* ox-ac. *petr.* *phos.* *ph-ac.* *plat.* plb. *puls.* ran-b. rheum. rhod. *rhus.* ruta. sabad. sabin. sars. *secale.* selen. sep. *sil.* spig. squil. stann. staph. *stram.* *sulph.* sul-ac. tabac. tanac. tarax. taxus. tereb. valer. verb. *verat.* verat-v. vesp. viol-od. vip. *znc.*
— **alternating** with convulsions: agar.
— — — dangerous violence: absinth.
— — — restlessness during fever: ars.
— **answers** correctly when spoken to, but delirium and unconsciousness return at once: arn. bapt. *hyos.*
— **interrupted** by screaming: bell.
— **dream**, as in a: ambr. carb-an. con. rheum. stram. valer. verat.
— does not know where he is: merc. nx-m. ran-b.
— — — — — — — on waking: æsc-h.

— **remains** fixed in one spot: nx-m.
— — motionless like a statue: hyos. stram.
— **sudden**: k-ca. plb.
— **transient**: bov. bufo. cann-i. canth. chel. hepar. nat-m. ol-an. rhus-r. sil.
— with tingling in head and limbs, better from motion: rhus.
— — tingling in head and limbs, worse from motion: secale.
— — single jerks: calad.
— — motion of feet: znc.
— — drowsiness in head, on rising in morning: rhod.
— — — afternoon in warm room: puls.

**Conditions** of Unconsciousness.
— **morning**: bov. carb-an. glon. lyc. nat-m. nx-v. phos. ph-ac. strych. sulph.
— — on waking: chel. nat-c.
— **evening**: acon. calc. caus. coloc. lyc. merl. nx-m. ol-an. strych. znc.
— — when lying down: mag-c. mag-m.
— **night**: arg-n. bell. cann-i. chel. nat-m.
— — on waking: con. mag-c. phos.
— **air**, in open: nx-v.
— — — amel.: tarax.
— **alone**, when: ph-ac.
— **candle** light, from: cann-i.
— **chill**, during: *bell.* camph. *hepar.* NAT-M. nx-v. op. puls.
— **cold** water poured over head, amel.: tabac.
— **convulsions**, with: absin. acon. ars. bell. cupr. glon. hydr-ac. stram. tanac.
— — after: carb-ac. secale.
— **delirium**, after: atrop.
— **diarrhœa**, after: ars.
— **dinner**, after: cast. tilia.
— **erect**, if he remained: chin.
— **excitement**, after: nx-m.
— **eyes**, with fixed: æth. ars. camph.
— — cannot open: gels.
— — with pressure in and obscuration of sight: seneg.
— **headache**, during: æth. agar. arn. *bov.* cann-i. cast. ferr. hepar. iod. phos. sil. stann. tarax.
— — and after: bov.
— — congestion to head, from: bell. hyos.
— — on moving: calc. carb-an. rhus.
— **heat**, with: æth. bell. laur. NAT-M. sol-n.
— **lying** down, when: colch. mag-c. *mag-m.*
— **menses**, before: nx-m.
— **music**, from: cann-i.
— **pain**, after: phyt.
— **reading**, from: asaf.
— **riding**, when: grat. sil.
— **rubbing** soles of feet, amel.: chel.
— **sitting**, when: asaf. carb-an. mosch. tarax.
— **somnolency**, with, without snoring, eyes being closed: ph-ac.
— **standing**, when: bov. lyc. rhus-r.
— **stooping**, when: calc.
— **suppression** of eruptions, after: znc.
— **talking**, when: lyc.
— **vertigo**, during: æth. agar. arg-n. arn. ars. bov. canth. carb-an. chel. chin-s. creos. ferr. grat. jatr. laur. lyc. mezer. mill. mosch. nat-m. nx-m. op. secale. sil. stram. tabac. znc.
— **vomiting**, amel.: acon. tabac. tanac.
— **waking**, on: æsc-h. chel. chin. mag-c. mezer. nat-c. phos.
— — after: con. k-bro. selen. stram.
— **walking**, when: calc. carb-an.
— — in open air: canth. caus. hepar. sulph.
— **yawning** and nausea, with: jatr.

**Undertakes** nothing, lest he fail: *arg-n.*
— things opposed to his intentions: sep.
— lacks will power to undertake anything: pic-ac.

**Unfeeling**: *anac.*

**Unfortunate**, feels: sep.

**Unfriendly** humor: am-c. mag-m. plat.

**Unpleasant** occurrences, likes to dwell on past: nat-m.

**Unworthy**, objects seem: *chin.*

**Verses**, makes: agar. ant-cr. cann-i. stram.
— after falling asleep: nat-m.

**Vexed**: *acon.* æth. agn. alum. ambr. am-c. am-m. anac. ang. ant-cr. ant-t. ars. asaf. aur. bar. bell. bov. *bry.* calc. cann-s. canth. caps. carb-v. caus. *cham.* chin. cocc. *coff.* coloc. con. cop. croc. fluor-ac. gamb. graph. hepar. ign. iod. ipec. iris. k-ca. lach. laur. led. *lyc.* mag-c. mang. marum. merc. *mezer.* nat-c. *nat-m.* nat-p. nitr. *nit-ac.* *nx-v.* par. petr. phos. ph-ac. plan. plat. ran-b. rhus. ruta. sabad. sabin. sars.

sep. *sil.* spig. spong. squil. stann. staph. sulph. sul-ac. verat. verb. znc.
— Compare with Ill-humor, Fretful, etc.
— **alternating** with cheerfulness: ant-t. borax. caus. cocc. croc. nat-m. spong.
— about **business**: ipec.
— **contradiction**, from: calc-p.
— **conversation**, from: puls. tarent.
— **pain**, at: op.
— in **sleep** by noise: calend.
— **trifles**, at: creos. nat-c. nat-p. sabad.
— **work**, about: nat-m.

**Violent**, vehement, etc.: abrot. acon. ambr. am-c. anac. ang. apis. arn. aur. bar. bell. borax. *bry.* calc. calc-p. camph. canth. carb-v. caus. *cham.* coloc. corn. croc. cupr. dros. dulc. ferr. graph. hepar. hyos. k-ca. lach. led. lyc. mang. merc. merl. mezer. mosch. nat-c. nat-m. nit-ac. *nx-v.* olnd. petr. phos. plat. ran-b. sabad. seneg. sep. stann. stram. stront. *sulph.* verat.
— Compare with Anger, Rage, Wildness, etc.
— **deeds**, rage leading to: anac. bar. *bell.* chin. cocc. con. hepar. hyos. *ign.* lach. lyc. mosch. nat-c. nx-v. plat. stram. stront. znc.
— in **morning**: carb-v. gamb. graph.
— **evening**: mill.
— after dinner: mill.
— after siesta: caus.
— at trifles: hepar. nat-m.

**Visions.** See Delirium, Delusions, etc.

**Vivacious**: alum. ang. cann-s. chin. *coff.* crotal. cupr. cyc. gels. glon. hipp. *hyos.* lach. nat-c. nx-v. par. petr. ph-ac. sabad. sul-ac. valer. verat.
— alternating with sorrow: tarent.

**Walking** rapidly from anxiety: arg-n.

**Wander**, desires to: calc-p. cimic. merc.
— See also Restlessness.

**Washing**, bathing, averse to: phys.
— desire for: tarent.
— — — in cold water: meph. phyt.

**Weariness** of life: agn. ambr. am-c. ant-cr. *ars.* AUR. bell. berb. bov. calc. carb-v. caus. chin. creos. grat. hepar. hyos. lach. led. lyc. merc. mezer. nat-c. nat-m. nit-ac. *nx-v.* phos. plb. puls. rhus. ruta. sep. spong. staph. stram. sulph. sul-ac. thu. valer. verat.
— in bed, morning: lyc.
— See Ennui, Loathing, etc.

**Weeping**, tearful mood, etc.: *acon.* alum. ambr. am-c. am-m. ang. ant-cr. ant-t. apis. arn. ars. asar. *aur.* aur-m. bar. *bell.* borax. *bry. calc.* camph. cann-i. cann-s. canth. caps. carb-an. carb-v. cast. *caus. cham.* chel. chin. chin-s. *cic. cina.** clem. cocc. *coff.* coloc. con. creos. *cupr.* cupr-ac. cyc. *dig.* dros. dulc. eup-per. eup-pur. *graph. hepar.* hyos. *ign. iod.* ipec. k-bro. k-ca. k-iod. lac-can. lach. lact. laur. led. lil-t. LYC. mag-m. mag-s. *mang.* men. merc. mezer. mosch. nat-c. NAT-M. nitr. nit-ac. nx-m. *nx-v.* op. *petr.* phel. phos. ph-ac. *plat.* plb. psor. PULS. ran-b. rheum. *rhus.* ruta. sabin. sars. secale. *sep.* sil. sol-n. spig. *spong.* stann. staph. stram. SULPH. sul-ac. tabac. tarent. thu. tilia. VERAT. *viol-od.* viol-tr. znc. ziz.
— Compare with Moaning, Crying out, etc.
— **aloud**: coff. hyos. nx-v. phos. plat. plect. sabin. staph. sulph.
— **alternating** with cheerfulness: acon. arg. bell. borax. cann-s. carb-an. ign. iod. plat. spong.
— — — laughter: *acon.* alum. aur. bell. caps. lyc. nx-v. plat. sep. stram. sulph. ziz.
— — — queer antics: cupr.
— — — irritability and laughter at trifles: graph.
— — — ill-humor: bell.
— **ameliorates** symptoms: *anac. dig. graph.* ign. *lyc.* merc. phos. plat. tabac.
— **inclination** to: acon. æth. alum. arg-n. ars. aur. *bell. bry. cact.* camph. canth. *carb-v.* cast. *caus. cham.* chel. cic. cimic. *cina.* cocc. coff. colch. croc. cupr. cyc. ferr. gins. *graph.* hæmat. hell. hura. hyos. ign. iod. ipec. k-ca. lact. laur. lil-t. merc. merl. *merc-i-r.* mezer. naja. nat-c. NAT-M. nat-s. *nit-ac.* nitr. *nx-m.* op. phos. *plat. puls.* ran-b. rheum. *rhus.* ruta. sabin. samb. *sep.* sil. spong. squil. *staph.* stram. sulph. sul-ac. tarent. thea thu. verat. znc.
— **involuntarily**: alum. *aur. bell.* cann-i. *caus.* cina. cupr. *ign.* lach. merc. mosch. NAT-M. *plat.* plb. rhus. stram. verat. viol-od.
— — amel. by vinegar: stram.
— **nervous**, feels so, she would scream. unless she held on to something: *sep.*

**Conditions** of Weeping.
— all day: bry. caus. lac-can. lyc.
— — feels like crying all the time, but it makes her worse: stann.
— **morning**: am-c. bell. borax. carb-an. creos. dulc. phos. plat. prun. puls. rhus. sars. sil. spong. sulph. tarent.
— — inclined to, at 11 A. M.: SULPH.
— — after waking: phos. pus. puls.
— **afternoon**: carb-v. cast. cop. dig. phos. tarent.
— **evening**: acon. *calc.* carb-an. clem. coca. graph. hyper. *plat.* rhus. sil. *stram.*
— — amel. in.: am-c. caus.
— — morning, and: sulph.
— **night**: alum. am-c. anac. arn. ars. bar. borax. bry. calc. carb-an. caus. cham. chel. *chin.* chin-s. *cina.* con. hipp. hyos. ign. ipec. k-ca. k-iod. lac-can. *lach.* lyc. mag-c. merc. *nat-m.* nit-ac. *nx-v.* op. phos. ph-ac. puls. rheum. sil. spong. stann. sulph. tabac. tarent. thu. verat.
— — in sleep: alum. carb-an. caus. cham. chin. con. ign. lach. lyc. nit-ac. *nx-v.* thu.
— — on waking: chin-s. sil.
— **admonitions**, cause: bell. calc. chin. ign. *k-ca.* lyc. nat-m. nit-ac. plat. staph.
— **air**, in open: carb-v. hura.
— — — amel. in: coff. *plat.*
— **alone**, when: con. nat-m.
— **anger**, after: arn. *nx-v.*
— — from: bell.
— **breathing**, from: bell.
— **carried**, when: chel.
— — child cries piteously if taken hold of or carried: *cina.*
— — is quiet only when carried: CHAM.
— **causeless**: apis. ars. bell. camph. *cina.* creos. graph. hura. lyc. *nat-m.* nit-ac. staph. sulph. tarent. viol-od. znc.
— — without knowing why: k-ca. rhus. sep.
— **contradiction**, from: tarent.
— **convulsions**, during: absin. cham. plb.
— **coughing**, before: *arn.* bell.
— — during: ant-t. arn. ars. *bell.* cham. chin. *cina.* HEPAR. ipec. lyc. osm. samb. sep. sil. spong. sulph. verat.
— — after: arn. bell. caps. cina. hepar. op.
— **delirium**, after: nat-s.
— **disturbed** at work, when: *puls.*
— **dreaming**, when: phos.
— **emission**, after an: hipp.
— **emotion**, after slight: creos. naja.
— **fever**, intermittent, during the chill: acon. ars. *aur.* BELL. CALC. cann-s. carb-b. CHAM. con. hepar. k-ca. LYC. merc. nat-m. *petr.* plat. PULS. selen. sil. sulph. VIOL-OD.
— — **heat**, during the: ACON. BELL. calc. *caps.* cham. coff. cupr. graph. ign. *lyc. petr.* plat. PULS. *spig.* SPONG. *stram.* sulph. tilia.
— — **sweat**, during the: acon. aur. BELL. bry. *calc. camph. cham.* chin. *cupr.* graph. LYC. nx-v. *op. petr. puls.* rheum. rhus. sep. spong. *stram.* sulph. verat.
— **food**, after: arg-n. arn. puls.
— **future**, about the: lyc.
— **hallucinations**, after: dulc.
— **headache**, with: coloc. creos. phos. plat. ran-b. sep.
— **illness**, during: calad.
— **joy**, from: lach.
— **laughter**, after: cann-s.
— — See Alternating with.
— — at amusing things: *plat.*
— **looked** at, when: *nat-m.*
— **mania**, during: ars.
— **menses**, before: cact. con. lyc. *phos. puls.* sep. *znc.*
— — **during**: ars. cact. calc. caus. con. cyc. hyos. *ign.* lach. lyc. phos. *plat. puls.* secale. sep. thu. verat. znc.
— — — which does her good: cyc.
— — **after**: alum. con. lyc. phos. stram.
— **micturition**, before: *borax. lyc. sars.*
— — during: erig. *sars.*
— **music**, from: creos. *graph.* nat-s. nitr. nx-v. thu.
— — of piano: cop.
— — of bells: ant-cr.
— **need**, about a fancied: chin.
— — about a past: nat-m.
— **nightmare**, after: guai.
— **noise**, at: æth. creos. ign. *lach.*
— **opposition**, at least: nx-v.
— **palpitation**, during: phos.
— **past** events: nat-m.
— **pains**, with the: glon. plat. puls.
— **pitied**, if believes he is: nat-m.
— **poetry**, at soothing: lach.
— **refused** anything, when: bell. cham. ign.

— **remonstrated** with, when: bell. calc. ign. *k-ca.* nit-ac. plat.
— **respiration**, with difficult: ant-cr. coca. thu.
— **rising**, after: am-c.
— **sad** thoughts at: alum. carb-v. cina. k-ca. phel. plat. stram.
— — — though sad, is impossible to weep: *nx-v.*
— **singing**, when: hura.
— **sleep**, in: all-s. alum. ars. bell. calc. carb-an. *caus. cham.* chin. con. creos. fluor-ac. glon. graph. hyos. k-ca. k-iod. lach. lyc. mag-m. *nat-m.* nicc. *nx-v.* phos. *puls.* rhus. *sil.* stann. sulph. tabac. tarent. thu.
— — good during the day, screaming and restless all night: *jalap.*
— **spoken** to, when: *nat-m.* plat. sil. *staph.*
— — kindly, soothing words, etc.: bell. calc. chin. ign. k-ca. nat-m. nit-ac. plat. staph.
— **stool**, before: phos. puls. rhus.
— — during: bell. *borax. cham.* cina. phos. rhus. sil. sulph.
— **taken** up, child, when: chel.
— **telling** of her sickness, when: PULS.
— **thanked**, when: lyc.
— **touched**, when: ant-cr. ant-t. cina.
— **trifles**, at: ant-cr. calc. caus. cina. cocc. petr. stram.
— — at the least worry, children: lyc. nit-ac.
— **vertigo**, with: phos.
— **vexation**, from: calad. cham. ign. nx-v. tarent.
— **wakens**, arouses from sleep: alum. cina. k-iod.
— **waking**, on: bell. chin-s. cina. hyos. ign. lach. lyc. merc. nicc. nx-v. op. phos. plan. puls. raph. sil.
— **walking** in open air, when: calc. coff.
— **writing**, when: coca.
— **weeks**, for: alum. mezer.
**Whistling**, inclination to: bell. cann-i. cann-s. carb-an. lyc. merc-i-fl. plat. stram.
— involuntarily: carb-an. lyc.
**Wildness**: acon. ant-t. bapt. calc-p. croc. mosch. op. petr. phos. ph-ac. *stram. verat.*
— See also Anger, Rage, Violent, etc.
— during headache: bapt.
— bright light, odors, etc.: *colch.*
— at trifles: ign.
— unpleasant news: calc-p.
— from vexation: ph-ac.
**Will**, contradiction of: acon. anac. caps. sep.
— want of control of: apis. lach. nat-m.
— deficient: *anac.* alum. *ars.* asaf. bar. bry. calc. camph. chin-s. cina. clem. coff. coloc. croc. dulc. ign. ipec. k-ca. lach. *merc.* nat-c. nat-m. op. petr. puls. rheum.
— strong: aloe. calc. camph. chin-s. plb.
— two, feels as if he had two wills: anac.
**Witty**: alcoh. caps. cocc. croc. *lach.* spong.
**Women**, aversion to: am-c. bapt. dios. nat-m. puls. raph.
**Work**, aversion for mental: acet-ac. acon. agar. aloe. anac. aur. bell. brom. *chin.* cinnb. clem. colch. ferr. grat. ham. hydr. hyper. iod. k-bi. lyc. mag-m. marum. meph. nit-ac. nitr. op. par. petr. phos. ph-ac. plat. ran-sc. rhus. rumx. *sil.* spig. squil. staph. sulph. thea. viol-tr.
— desire for mental: brom. clem. cob. coca. naja. rhus. therid.
— complaints from: lyc. nat-m.
— compare Work in general.
**Writing**, aversion to: hydr. squil. thea.
— **desire** for: chin. spig.
— **difficulty** in expressing ideas, when: carb-an.
— **inability** for: ign. lyc.
— — — writing connectedly: colch.
— **mistakes**, makes, in: am-c. benz-ac. bov. calc-p. cann-i. cann-s. carb-an. cham. chin. chin-s. chrom-ac. croc. crotal. dios. fl-ac. graph. hepar. hydr. ign. k-bro. lac-can. lach. lac-ac. *lyc.* nat-c. nat-m. nx-m. nx-v. ptel. puls. rhod. rhus. sep. sil. *thu.*
— — **adding**, in: lyc.
— — **omitting** letters, by: hyper. k-bro. lac-can. lyc. nx-v. puls. stram.
— — omitting words, by: cham. k-bro. lyc. nx-v. thu.
— — **repeating** words, by: calc-p. k-bro. lac-can.
— — **transposing** letters: stram.
— — **wrong** words, by using: am-c. calc-p. cann-i. *lyc.* sep.
— — by putting "right" for "left," or *vice versa:* chin-s. fluor-ac.
**Wrong**, everything seems: coloc. eugen. hepar.

